# Development of methyl 5-((cinnamoyloxy)methyl)picolinate, exploring its bio-target potential aiming at CVD mediated by multiple proteins through surface and physiochemical analysis

**DOI:** 10.1038/s41598-024-64165-7

**Published:** 2024-06-10

**Authors:** Lenin Nachimuthu, Rajagopal Desikan

**Affiliations:** Department of Chemistry, School of Advanced Sciences (SAS), Vellore Institute of Technology (VIT), Vellore, 632014 Tamilnadu India

**Keywords:** Cinnamic acid, Density functional theory, Molecular docking, Swiss ADME, Cardiovascular diseases, Structural biology, Biomarkers, Chemistry

## Abstract

The emphasis on sustainable sources of drug development seems imminent with phytochemicals emerging as promising candidates due to their minimal probability of adverse effects. This study focuses on utilizing simple cinnamic acid and nicotinic acid derivatives as starting materials, employing an efficient synthetic protocol to obtain methyl 5-((cinnamoyloxy)methyl)picolinate targeting CVD mediated by multiple enzymes such as MAPK, PCSK9, MPO, SIRT1 and TNF-α. Comprehensive characterization of synthesized molecule is achieved through ^1^H, ^13^C, FT-IR, and HRMS methods. Additionally, the crystal structure was established via SC-XRD. Comparative analysis with the DFT-optimized structure identifies key nucleophilic and electrophilic regions for determining interactions with bio-targets. Notably, Compound 5 adheres to all drug-likeness criteria, further validated through screening similar pharmacophoric drugs from databases. Targeting bio-relevant areas with a specific focus on CVD drug development. The molecular docking studies elucidate ligand–protein interactions for better binding connectivity. This investigation further underscores the importance of sustainable practices, simple chemical synthesis, and computational approaches, contributing to the pursuit of eco-friendly drug development with enhanced safety profiles (MTT assay).

## Introduction

**Figure 1:**
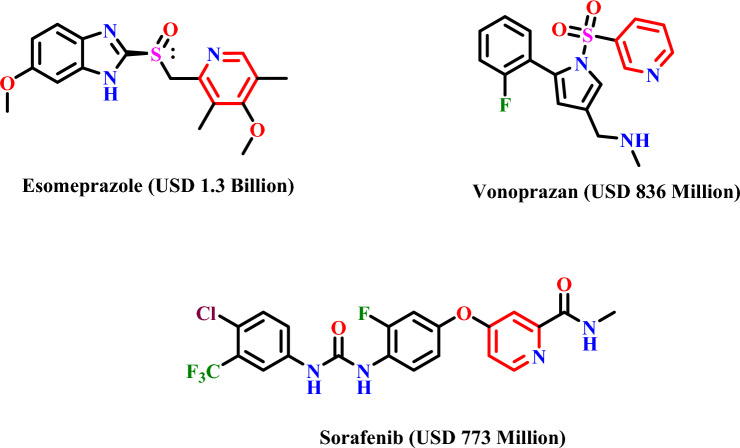
Chemical structures of nicotinic scaffolds containing top-selling pharmaceutical small molecules in 2022.

Nicotinic acid, commonly known as niacin, is a vital phytochemical integral to daily human life. Typically, several types of nicotinic acid-containing drugs have been developed to elevate the HDL (high-density lipoprotein)-cholesterol and earn FDA approval for treating dyslipidemia, as well as to reduce LDL (low-density lipoprotein)-cholesterol, LPa (lipoprotein-a), and VLDL (very low-density lipoprotein)-cholesterol levels in blood plasma^1–3^. In addition, nicotinic acid scaffolds play a widespread role in the synthesis of biomolecules. Generally, nicotinic acid is found in vitamin B3 and vitamin B6 and serves as precursors for nicotinamide adenine dinucleotide (NAD+) and nicotinamide adenine dinucleotide phosphate (NADP+) coenzyme synthesis^4^. The pyridine moiety plays a significant role in regulating the heart's intermediate metabolism, whereas the third-position carboxylic group in nicotinic acid plays a crucial role in the bioactivities of these phytochemicals. The presence of a modifiable group serves as a pivotal functional pharmacophore motif, enhancing the versatility of chemical building blocks^5^. Notably, small-molecule drugs featuring nicotinic scaffolds attained remarkable sales in 2022, (Fig. 1) underscoring their pharmaceutical importance^6^.

However, nicotinic acid is recognized to have adverse effects that restrict its clinical usage, even though all of these have positive therapeutic advantages. Moreover, side effects include gastrointestinal (GI) problems, and flashing sensations in the face and upper body^7–9^. Therefore, it is necessary to modify the structural aspects of nicotinic acid. On the other hand, renowned phytochemicals are caffeic acid, coumaric acid, ferulic acid, and sinapic acid, which are prevalent and present in diversified plants and contain the core structure of cinnamic acid. Traditionally employed in medicinal preparations due to their accessibility and potency, cinnamic acid-type molecules are now subject to derivatization for better active phytochemical derivatives. Specifically, cinnamic acid has garnered attention for its modified forms, revealing antimicrobial, antibacterial, and antiproliferative effects^10,11^. Cinnamic acid-containing cinnamon plants deliver promising therapeutic effects towards cardioprotective activity. Therefore, it is logical to utilize both entities for the benefit of creating new structures that would show synergism in the bioactivity in a single molecule. For this synergism, there is a need to couple nicotinic acid and cinnamic acid to create a better-acting entity^12,13^.

In this study, the chemical combination of nicotinic and cinnamic scaffolds is achieved through organic synthesis, followed by compound purification and the subsequent acquisition of a single crystal for the final molecular entity. This novel compound represents a pioneering fusion of two distinct phytochemical scaffolds, potentially introducing a novel avenue for synergistic effects. Comprehensive investigations ensue, involving various characterization techniques, with data meticulously compared against results obtained from quantum calculations. Surface interactions of the crystal are scrutinized, unveiling insights into its unique properties. Lead likeness attributes are then assessed, revealing structural similarities to established cardiovascular and lipid-lowering agents. Molecular docking studies probe the interactions between the synthesized molecule and crucial biomarkers associated with lipids and cardiovascular diseases. The analytical approach encompasses a diverse array of techniques, delving into both surface and physical parameters to provide a thorough examination of the synthesized molecule's properties and potential therapeutic implications.

## Materials and methods

### General techniques

The starting materials and reagents are purchased from Sigma-Aldrich, TCI Chemicals, and Merk. For the structural analysis, Bruker 400 MHz was used for analyzing the ^1^H and ^13^C NMR of the synthesized compound. UV absorption studies Jasco V-670 spectrophotometer was employed for UV spectroscopy studies.

### Synthesis and re-crystallization of the title compound 5

#### Synthesis of dimethyl pyridine-2,5-dicarboxylate, compound 2

In a well-dried 100 mL round bottom (RB) flask equipped with nitrogen, 2,5-pyridine carboxylic acid (1.0 g, 0.006 mol) and methanol (10 v) were added and stirred for 10 min at 0–5 °C. Then, thionyl chloride (0.013 mol) was added dropwise into the above-stirred reaction mixture, and the entire reaction mass was refluxed for 3 h. After, reaction progression was observed by thin-layer chromatography (TLC). Following the complete conversion of the starting materials, methanol (4 v) was added and further stirred for 30 min at 30–35 °C. Then it was transferred into ice-cold water (20 v) and the compound was extracted using DCM (15 v × 2 times). The organic layer was washed with bicarbonate (10 v) and water (10 v), followed by brine solution (10 v). The Na_2_SO_4_ was added to the organic solution and concentrated under reduced pressure. A 72% yield of the light-yellow solid weighing 0.84 g was obtained (Table 1)^14^.

**Table 1:** Optimization of the reaction condition for increased yield Compound **2**.

S.no.	Reagent	Solvents	Reaction conditions	Yield (%)
1	H_2_SO_4_	Methanol	25–70 °C, 18 h, N_2_	68
2	SOCl_2_	Methanol	0–70 °C , 14 h, N_2_	72
3	SOCl_2_	Methanol	0 °C—reflux, 3 h, N_2_	72
4	(COCl)_2_, DMF	DCM, methanol	RT—3 h, 0 °C—20 min	65

#### Synthesis of methyl 6-(hydroxymethyl)nicotinate, compound 3

In a dried 3-neck 100 mL RB flask, a mixture of THF and methanol (30 v) was added in a 1:2 ratio. Compound **2** (0.5 g, 0.0026 mol) was added, and 0 °C was maintained. After 5 min, to this suspension, calcium chloride (1.11 g, 0.01 mol) was added and stirred for 30 min. In continuation, NaBH_4_ (0.25 g, 0.0065 mol) was added in nitrogen condition; this reaction was allowed it to proceed for 2 h at the same temperature. The reaction mass was monitored by TLC to confirm the starting material conversion, followed by the reaction mass being quenched with water (25 v). The compound was then extracted with chloroform (10 mL × 2). The organic layer was washed with water (10 mL), and brine (10 mL) was dried with Na_2_SO_4_ and concentrated under reduced pressure. The product was isolated by employing column chromatography and obtained as 0.38 g of the off-white solid with an 89% yield^15^.

#### Synthesis of methyl 5-((cinnamoyloxy)methyl)picolinate, compound 5

**Figure 2:**
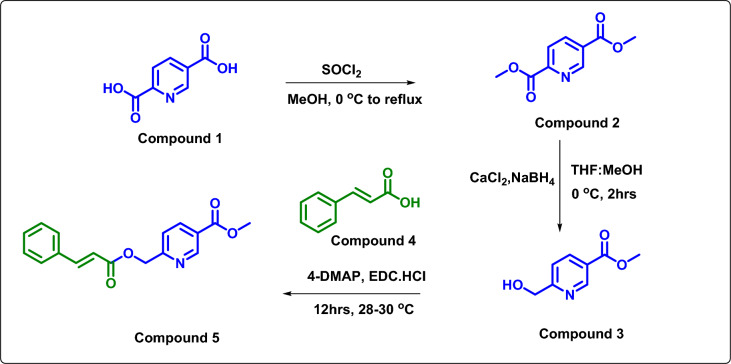
Synthesis Scheme of compound-5 preparation.

In the nitrogen condition, compound **3** (0.2 g, 0.001 mol) and compound **4** (cinnamic acid) (0.001 mol) were taken into a 25 mL RB flask containing dichloromethane (10 v) at 25 °C. In continuation, 4-dimethylaminopyridine (0.002 mol) was added and stirred for 30 min. Next, N-(3-dimethylaminopropyl)-N′-ethyl carbodiimide hydrochloride (0.002 mmol) was added and maintained at 28–30 °C for 12 h. The reaction mass was analyzed by TLC to confirm to the starting material conversion. After completion, the reaction mass was poured into 20 mL of water and extracted using DCM (15 mL × 3). The organic portion was separated and washed with water and brine solution. The Na_2_SO_4_ was added to an organic solution and concentrated under reduced pressure. The product was isolated by employing column chromatography and obtained 0.252 g of white solids with a 71% yield (Table 2). Then, pure compound **5** (Fig. 2) was tried for recrystallization using various solvents and ended up with transparent single crystals from the mixture of dichloromethane: diethyl ether (9:1) solvent at 25–35 °C via slow evaporation^16^.

**Table 2:** Optimization of the reaction condition for increased yield compound **5**.

S.no.	Base	Catalyst	Solvents	Reaction conditions	Yield (%)
1	DMAP	DCC	DMF	25–30 °C,12 h, N_2_	61
2	DIPEA	DIC	CH_3_CN	25–30 °C, 12 h, N_2_	52
3	TEA	BTC	DCM	25–30 °C, 12 h, N_2_	54
4	**DMAP**	**EDCI. HCl**	**DCM**	**25–30 °C, 12 h, N**_**2**_	**71**
5	DIPEA	EDCI, HOBt	DMSO	25–30 °C, 12 h, N_2_	56
6	TEA	DCC	Benzene	30 °C, 12 h, N_2_	60

Yield: (0.211 g) 72%, color: crystalline white, m.p.:106–107 °C. FT-IR (cm^−1^): 3076, 2928, 1729, 1280, 1160 (Fig. S5). ^1^H NMR (400 MHz, CDCl_3_) *δ*: 9.20 (s,1H), 8.32–8.30 (d, *J* = 8,1H), 7.81–7.77 (d, *J* = 16, 1H), 7.55–7.39 (m, 6H), 6.58–6.54 (d, *J* = 16, 1H), 5.43 (s, 1H), 3.96 (s, 1H), ppm. ^13^C NMR (100 MHz, CDCl_3_) *δ*: 166.40, 165.53, 160.43, 150.65, 145.99, 137.92, 134.20, 130.59, 128.96, 128.22, 125.17, 120.88, 117.22, 66.36, 52.43, ppm. HRMS (ESI) (Fig. S4): Molecular formula C_17_ H_15_NO_4_ [M + H], calculated mass = 298.1079 obtained mass = 298.1050.

### Single crystal X-ray diffraction analysis

A transparent single crystal of Compound 5 was analyzed for the X-ray data collection. The 0.110 mm × 0.140 mm × 0.260 mm size crystal study was carried out by Bruker Kappa APEX-II, and structure evaluation was done by the APEX 4 software. The graphite-monochromated Mo-kα radiation (λ = 0.71073 Å) was employed at 300 K for the data collection. The collected data was solved by APEX4 software, and after the refinement process, the R-factor value was 0.0473. This value relies on good-quality crystal; this structure was deposited in the Cambridge crystallographic data center, and the number is 2,221,434 (Table 3).

**Table 3:** Crystal results and structure refinement details for compound **5**.

Identification code	Shelx
Molecular formula	C_17_H_15_NO_4_
Molecular weight	297.30
Temperature	300 (2) K
Wavelength	0.71073 Å
Crystal system, space group	Triclinic, P-1
Unit cell dimensions	a = 6.1420(7) Å, alpha = 100.862(4) deg
	b = 7.5357(9) Å, beta = 91.039(4) deg
	c = 17.635(2) Å, gamma = 112.819(4) deg
Volume	735.10 (15) Å^3^
Z, calculated density	2, 1.343 g/cm^3^
Absorption coefficient	0.096 mm^-1
F(000)	312
Crystal size	0.110 × 0.140 × 0.260 mm
Theta range for data collection	2.98 to 28.29 deg
Limiting indices	− 8 ≤ h ≤ 8, − 10 ≤ k ≤ 10, − 23 ≤ l ≤ 23
Reflections collected/unique	20,269/3619 [R(int) = 0.0405]
Completeness	99.2%
Absorption correction	Semi-empirical from equivalents
Max. and min. transmission co-efficient	0.9750 and 0.9890
Refinement method	Full-matrix least-squares on F^2^
Data/restraints/parameters	3619/0/200
Goodness-of-fit on F^2^	1.035
Final R indices [I > 2 sigma(I)]	R1 = 0.0470, wR2 = 0.1082
R indices (all data)	R1 = 0.0759, wR2 = 0.1266
Largest diff. peak and hole	0.180 and − 0.163 e. Å^−3^

### Computational studies

After confirming the crystal structure of Compound **5**, the quantum chemical structural characteristics are analyzed using the Gaussian software version 16 by using B3LYP/6-311G^++^ basis sets for these operations^17^. From the optimized crystal structure, molecular geometry and molecular orbital energy levels (HOMO and LUMO) are analyzed using gas phase mode. Then the TD-DFT was performed, which compared the UV absorption values and correlated practically. Also, structural parameters such as dipole moment, electrostatic potential, and chemical potential are calculated. In continuation, the physical features of compound **5**, like intermolecular distance and Hirsh field studies, are analyzed by *Crystal Explorer* version 17.5. The crystal packing image was derived using Avagadro software version 1.2.0^18^. After confirming the complete structural characterization by theoretical as well as empirical methods, compound **5** was used to run and identify the biological responses toward the biotarget of oxidative stress. Primarily, compound **5** was run in the *SwissADME* database and collected the ADMET and lead likeness^19^. Then compound **5** was investigated for its binding interaction and energy with the five different biomarkers collected from the RCSB PDB database; PDB codes are 4FA2, 2P4E, 4I5I, 2AZ5, and 5FIW (two cholesterols, two diseases, and one oxidative stress), and the molecule-ligand binding interactions were investigated using *Auto dock tool 1.5.7*^20^. Then the interaction was visualized by the *Chimera X* and *Discovery studio visualizer* applications.

### MTT cell viability assay

The MTT assay was employed to examine the toxicity of the pyridine-cinnamic acid-fused heterocyclic molecule. RAW macrophage cells (RAW 264.7 from the ATCC) and different concentrations of the synthesized molecule were used for analysis in the MTT assay. This protocol is adopted by the previous literature^21^.

## Result and discussions

### Synthesis and structural characterization of cinnamic-nicotinic fused molecule

In this work, we report a new organic molecule containing cinnamic and nicotinic fragments, yielding 71%. Compound **5** is thoroughly characterized using NMR spectroscopy, IR spectroscopy, and HRMS spectrometry techniques.

The ^1^H NMR spectrum (Fig. S1) of compound 5 reveals a doublet of doublet pattern for the alkene bond, appearing in the range of 7.8 to 6.5 ppm. These two peaks exhibit a J value of 16, indicating the *trans*-isomeric form of the molecule. The aliphatic methylenic CH_2_ group is observed at 5.4 ppm, while the methoxy peak is detected at 3.9 ppm.

In the ^13^C NMR spectrum (Fig. S2), two carbonyl carbons are evident at 166 and 165 ppm, and aliphatic carbons corresponding to the methoxy and methylenic groups appear at 52 and 63 ppm, respectively. Symmetric carbons within the cinnamic acid phenyl ring manifest as two peaks around 128 ppm.

### Gas chromatography-mass spectrometry (GC–MS)

The structural fragmentations of compound 5 were analyzed by GC–MS (Fig. S3). The molecular mass of the compound is 297.32. In continuation, the fragment pattern was observed from the spectra. The fragmentation pattern was well in agreement with the bond dissociation energy, with pyridinyl (77.02) and styrene radical (103.05) fragments at the highest, and then the ester bond energy is low, which produces the cinnamic fragment (131.05) and pyridine fragment (166.05), which divide the whole molecule into two halves. Then the same case continues for the next fragment, which forms a dicarboxylate radical (179.02). The fragmented molecule structures are visualized with molecular mass values derived (Fig. S9).

### Study of single-crystal X-ray diffraction

Based on the crystallographic information provided and the chemical formula (C17H15NO4), here’s a description of the molecule’s structure: The molecule contains 17 carbon atoms (C), 15 hydrogen atoms (H), 1 nitrogen atom (N), and 4 oxygen atoms (O). The unit cell parameters indicate a triclinic crystal system with the P-1 space group. The cell dimensions are:

$$ \begin{aligned} {\text{a }} & = { 6}.{142}0\left( {7} \right){\text{\AA}} \\ {\text{b }} & = { 7}.{5357}\left( {9} \right){\text{\AA}} \\ {\text{c }} & = { 17}.{635}\left( {2} \right){\text{\AA}} \\ \end{aligned} $$

The cell angles are:

$$ \begin{aligned} {\text{alpha }} & = { 1}00.{862}\left( {4} \right){\text{ deg}} \\ {\text{beta }} & = { 91}.0{39}\left( {4} \right){\text{ deg}} \\ {\text{gamma }} & = { 112}.{819}\left( {4} \right){\text{ deg}} \\ \end{aligned} $$

**Figure 3:**
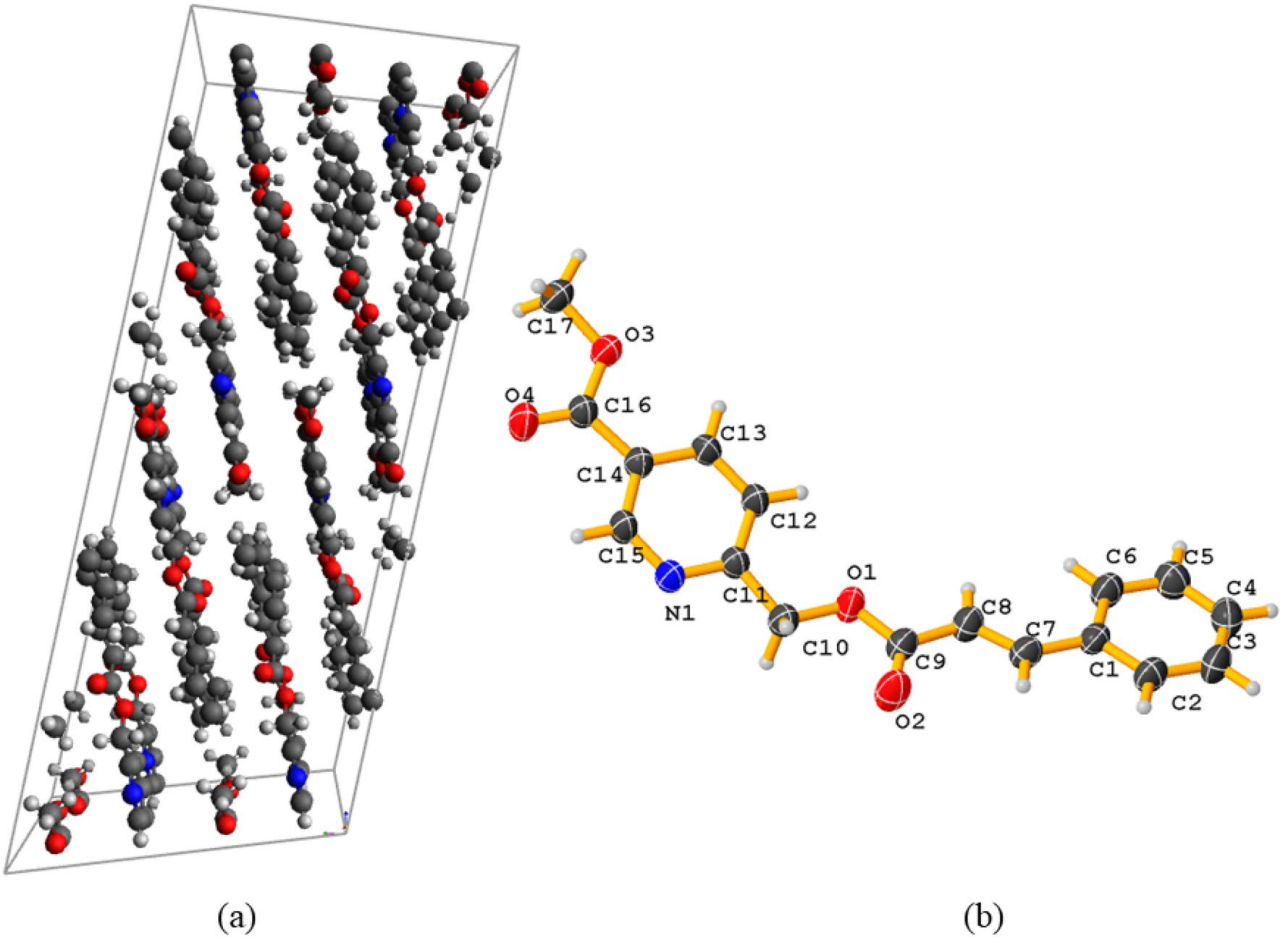
(**a**) Molecule arrangement in the unit cell, (**b**) Single crystal structure of compound 5 with the atomic label.

The calculated density is 1.343 g/cm^3^. The presence of nitrogen (N) suggests the molecule contains a heterocyclic aromatic ring. The four oxygen atoms (O) could be part of various functional groups, such as carbonyls (C=O) and esters. Within the molecule, bond angles and lengths will vary depending on the specific atom types and functional groups present. For example, C–C single bonds typically have a bond length of around 1.54 angstroms and a tetrahedral angle (around 109.5°). In contrast, C=O double bonds have shorter lengths (around 1.22 Å) and a more linear geometry (around 180°). Single bonds often allow for rotation, impacting the conformation of the molecule. The least symmetry is observed; this is the unique characteristic of the triclinic system. The packing structure of the molecule and the 3D arrangement of the molecule are provided in Fig. 3.

### Hirshfield surface investigation

**Figure 4:**
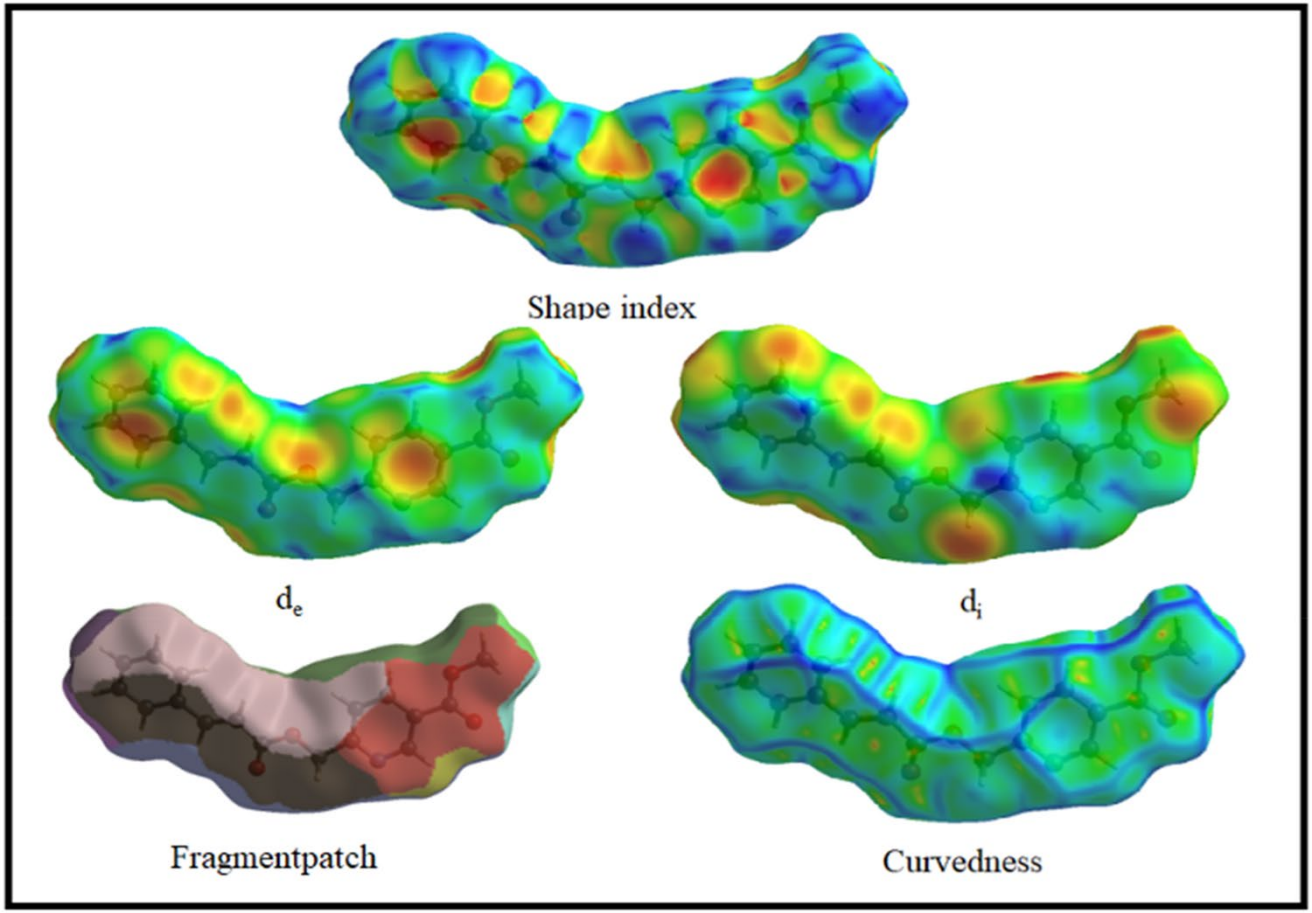
Surface image of the Shape Index, d_e_, d_i_, fragment patch, and curvedness.

**Figure 5:**
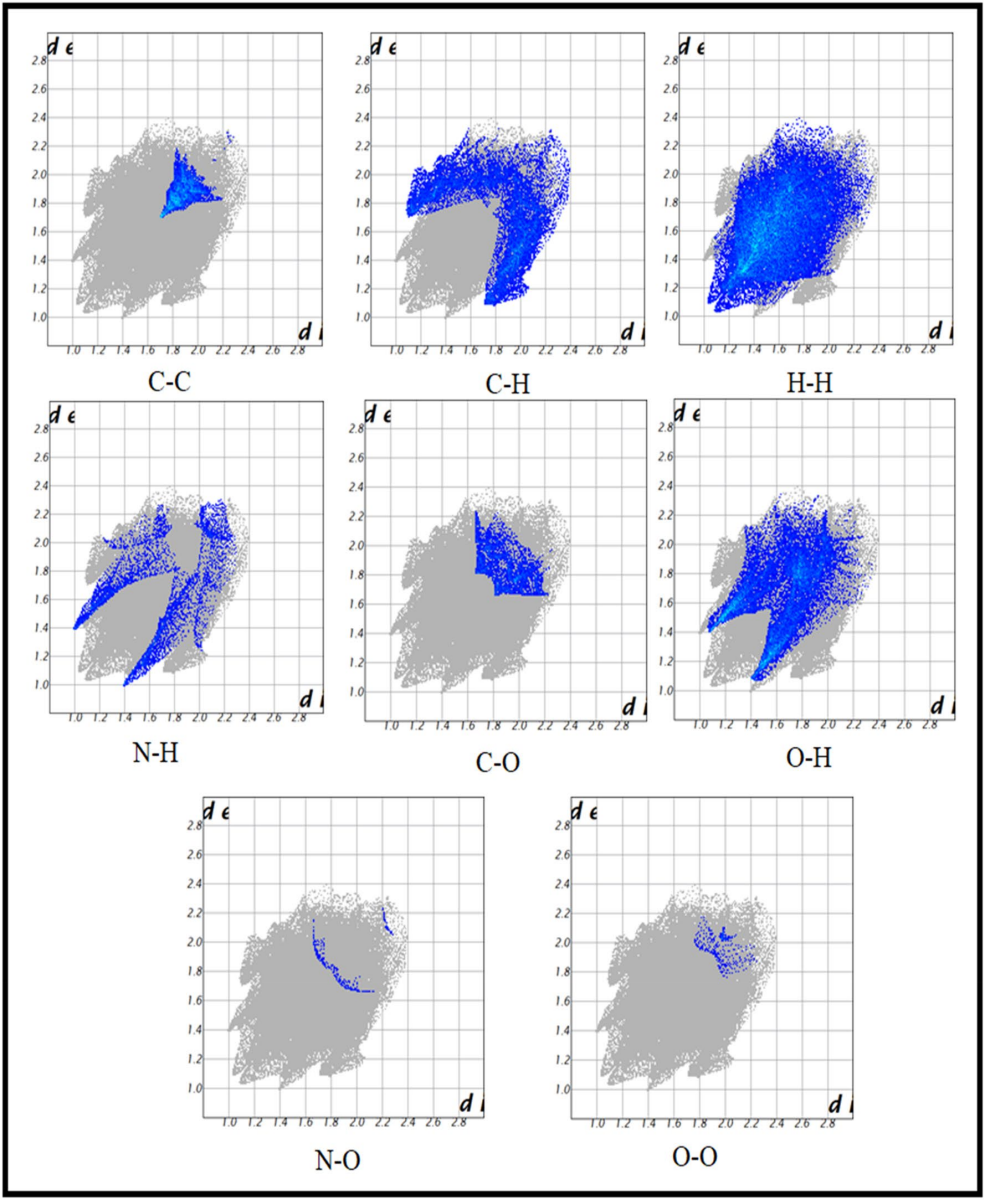
Structure of two-dimensional contact fingerprint plots.

The intermolecular interaction of the system was derived from the Hirshfield surface. The newly synthesized compounds need to determine the surface interactions involved in their physiochemical properties^22^. The Hirshfeld surface of compound 5 was analyzed using the *CRYSTAL EXPLORER 17.5* program^23^. The color patterns are used to portray the different intermolecular interactions on the Hirshfield surface. The functions are calculated by comparing the Hirshfield surface to the nearest nucleus inside the surface (d_i_) and outside the surface (d_e_). The d_e_ and d_i_ are both combined after each is normalized by van der Waal’s radii to produce the d_norm._ These maps are analyzed for the molecule, given in various parameters (shape index, d_e,_ d_i_, fragment patch) in Fig. 4. In the pyridine ring, the nitrogen has intermolecular hydrogen bonding with the next atom of the pyridine ring. This N⋯H–C interaction value is 2.406 Å. Another interaction involves a carbonyl group (C=O) with alkene hydrogen in the double bonds; this C–H^…^O value is 3.227 Å (Fig. S8). The two-dimensional contacts of the crystal are represented by fingerprint plots (Fig. 5). It depicts the H^…^H connections, including reciprocal contacts, which account for 44.9% of the total Hirshfeld surface map. The percentages of O^…^H, C^…^H, C^…^C, N^…^H, C^…^O, O^…^O, N^…^O, and N^…^C are 20.1%, 17.5%, 6.0%, 5.7%, 4.9%, 0.5%, 0.3%, and 0.1%, respectively. The intermolecular interactions within the structure of Compound **5** decoded the possibilities of bonding interactions with biological targets, which will increase the success rate for biological activities.

### DFT calculation

Quantum chemistry calculations are useful for the precise prediction of the structural and electronic features of molecules, like structure optimization, HOMO and LUMO energy barriers, TD-DFT UV spectra, molecular electrostatic potential, etc.

#### Structure optimization

The crystal structural geometry of compound 5 was optimized by using the B3LYP/6-31++(d,p) Gaussian basis set. The theoretical data on bond length and bond angle are provided in Table 4. The DFT analysis and single crystal XRD data were compared, and it was observed that most of the parameters were similar to each other. For example, in carboxylic acid C=O, the C9**–**O2 and C16**–**O4 theoretical bond lengths are 1.2148 Å and 1.2133 Å respectively. The experimental data obtained from the crystal structure is 1.1964 Å and 1.2010 Å for the same bonds (Fig. S6). The C**–**O bonds present in the crystal are such that the C9**–**O1 and C16**–**O3 theoretical bond length values are 1.3620 and 1.3525, respectively. Here, phase deviations are noticed because of theoretical calculations in the gaseous state and experimental analysis performed in the solid state. Significantly, theoretical data agreed with the real-time experiments.

**Table 4:** Experimental and theoretical bond length (Å) and bond angles (^o^) of the crystal structure of compound **5**.

Parameter	Experimental	DFT (calculated)
	Bond length (Å)	
O1–C9	1.3552	1.3620
O1–C10	1.4298	1.4369
O2–C9	1.1964	1.2148
O3–C16	1.3330	1.3525
O3–C17	1.4443	1.4407
O4–C16	1.2010	1.2133
N1–C15	1.3342	1.3339
N1–C11	1.3377	1.3436
C1–C6	1.388	1.4100
C1–C2	1.3928	1.4084
C1–C7	1.4627	1.4661
C2–C3	1.378	1.3959
C3–C4	1.367	1.3972
C4–C5	1.375	1.4011
C5–C6	1.378	1.3927
C7–C8	1.3186	1.3492
C8–C9	1.463	1.4743
C10–C11	1.4979	1.5091
C11–C12	1.3817	1.4007
C12–C13	1.3787	1.3930
C13–C14	1.3826	1.4003
C14–C15	1.3847	1.4039
C14–C16	1.4864	1.4890
	Bond angles (^o^)	
C15–N1–C11	117.47	118.10
C6–C1–C2	117.69	118.21
C3–C2–C1	120.88	120.69
C4–C3–C2	120.40	120.36
C3–C4–C5	119.74	119.66
C5–C6–C1	121.09	121.12
C8–C7–C1	127.27	127.22
O2–C9–O1	122.32	122.81
O1–C10–C11	109.69	109.74
N1–C11–C12	122.59	122.82
C13–C14–C15	117.75	117.85
C13–C14–C16	123.35	123.48
C15–C14–C16	118.89	118.66
N1–C15–C14	123.92	123.57
O4–C16–O3	123.41	123.16
O4–C16–C14	124.72	124.54

#### Frontier molecular orbitals

It represents the HOMO and LUMO energy levels of the molecules, and from this, the electron-transferring behaviour of the molecule will be identified. The HOMO is the ability to donate the electron. LUMO is the ability to accept an electron. Furthermore, HOMO represents the ionization potential (IP), and LUMO represents the electron affinity (EA) of the molecule. The characteristics can be calculated by Koopman’s theorem^24^. The formulas are given below:

1 $$ IP \, = \, - E_{HOMO} $$

2 $$ EA \, = \, - E_{LUMO} $$

The band gap between:

3 $$ \Delta E_{Gap} = \, \left( {E_{LUMO} - \, E_{HOMO} } \right) $$

The absolute chemical hardness (η) corresponds to the band gap, ΔE_Gap_;

4 $$ \eta \, = \, \left( {IP \, {-} \, EA} \right)/2 \, = \, \Delta E_{Gap} /2 $$

The electronegativity (χ):

5 $$ \chi \, = \, \left( {IP + EA} \right)/2 $$

The softness (σ):

6 $$ \sigma \, = \, 1/ \, \eta $$

The chemical potential (μ_p_):

7 $$ \mu_{p} = \, - \, \left( {IP \, + \, EA} \right)/2 $$

The electrophilicity index (ω)

8 $$ \omega \, = \, \mu_{p}^{2} /2\eta $$

**Figure 6:**
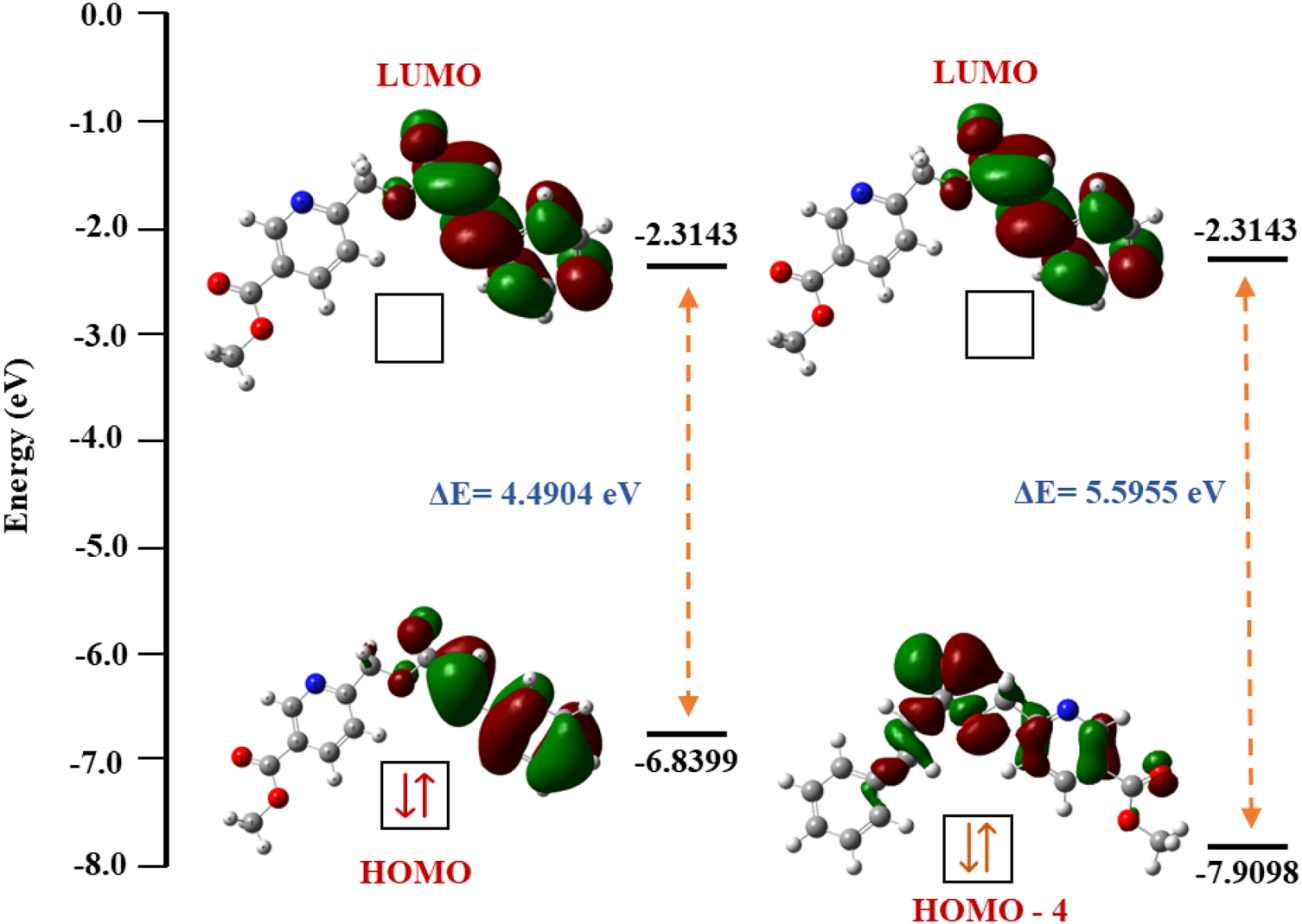
HOMO and LUMO surface cloud map for compound **5** with an energy score.

The optimized structure was used to study the frontier molecular orbitals. From that, HOMO and LUMO surface images are present in Fig. 6. The obtained energy values of HOMO and LUMO are − 6.8399 eV and − 2.3143 eV, respectively. After that, Koopman’s theorem implied that various chemical parameters like electronegativity (χ), chemical hardness (η), softness (σ), chemical potential (μ_p_), and electrophilicity index (ω) were evaluated. The quantum chemical parameter data is provided in Table 5. The HOMO and LUMO transitions are involved in the cinnamic part of the molecule.

**Table 5:** Quantum chemical parameters of the compound **5**.

DFT analysis	Energy
E_TOTAL_ (Hartree)	− 1012.556724
Dipole moment	6.4878 debye
*E*_(HOMO)_	− 6.8399 eV
*E*_(LUMO)_	− 2.3143 eV
∆E_Gap_ (HOMO–LUMO)	4.5256 eV
Electronegativity (χ)	4.5771
Chemical potential (μ_p_)	− 4.5771
Global hardness (*ƞ)*	2.2628
Softness (σ)	0.4419
Global electrophilicity index (ω)	4.6292

#### Molecular electrostatic potential

**Figure 7:**
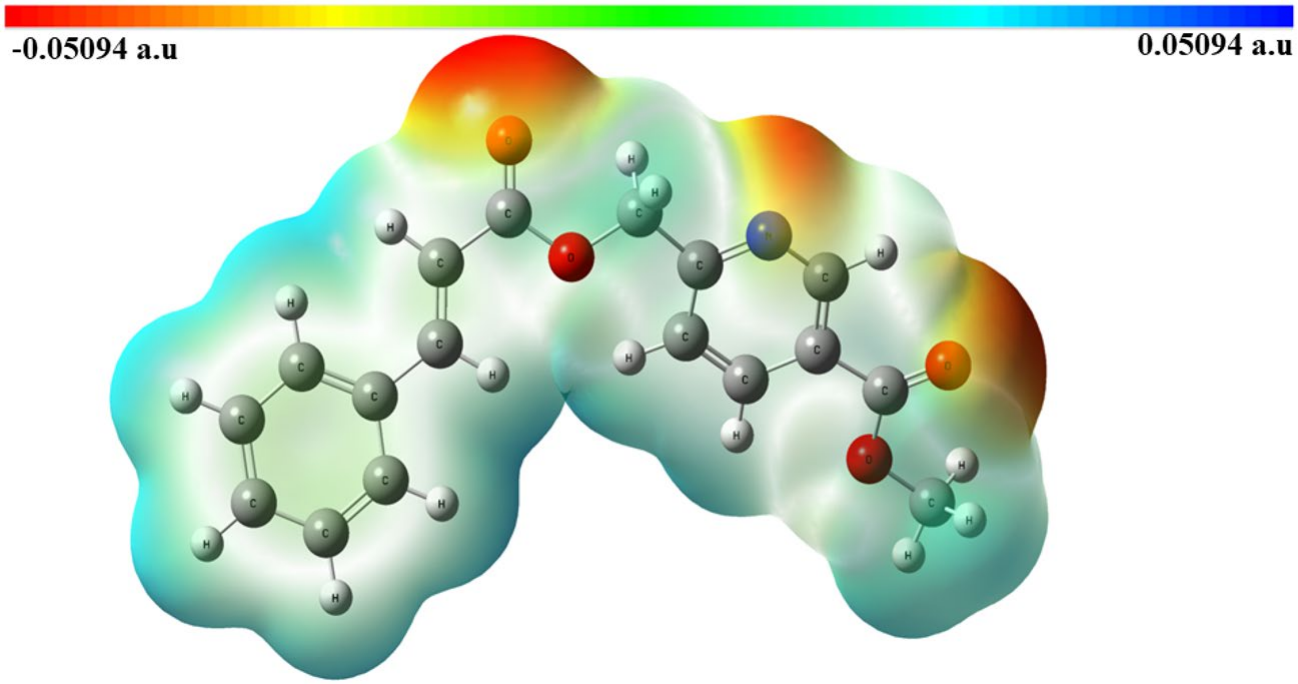
Molecular electrostatic potential surface cloud map for compound **5**.

The molecular electrostatic potential is one of the tools for determining the surface electronic features of the molecule’s intensity of charges, nuclei, and the exact position of the electron. It is also useful to predict non-covalent interactions, which is necessary for the pharmacophore to depict the interaction with the nucleophilic and electrophilic amino acid sites. This will be useful for the prediction of the molecule’s interactions with the protein. The optimized structure is evaluated for the MEP from the 3D map of the molecule's surface electrostatic potential derived for the identification of different color codes used for the map. The region that is preferred for electrophilic attacks and has a high electron density is indicated in red. The region that is preferred for nucleophilic attacks and has a low electron density is shown in blue. Figure 7 shows the MEP surface cloud map image. These MEP values are in the range of − 0.05094 to 0.05094 a.u. The cinnamic acid side acts as the nucleophilic, and the nicotinic scaffold atoms act as electrophilic charge transfer in the bioactivity of the molecule. The charge separation is properly divided in the crystal molecule^25^.

### UV absorption study

**Figure 8:**
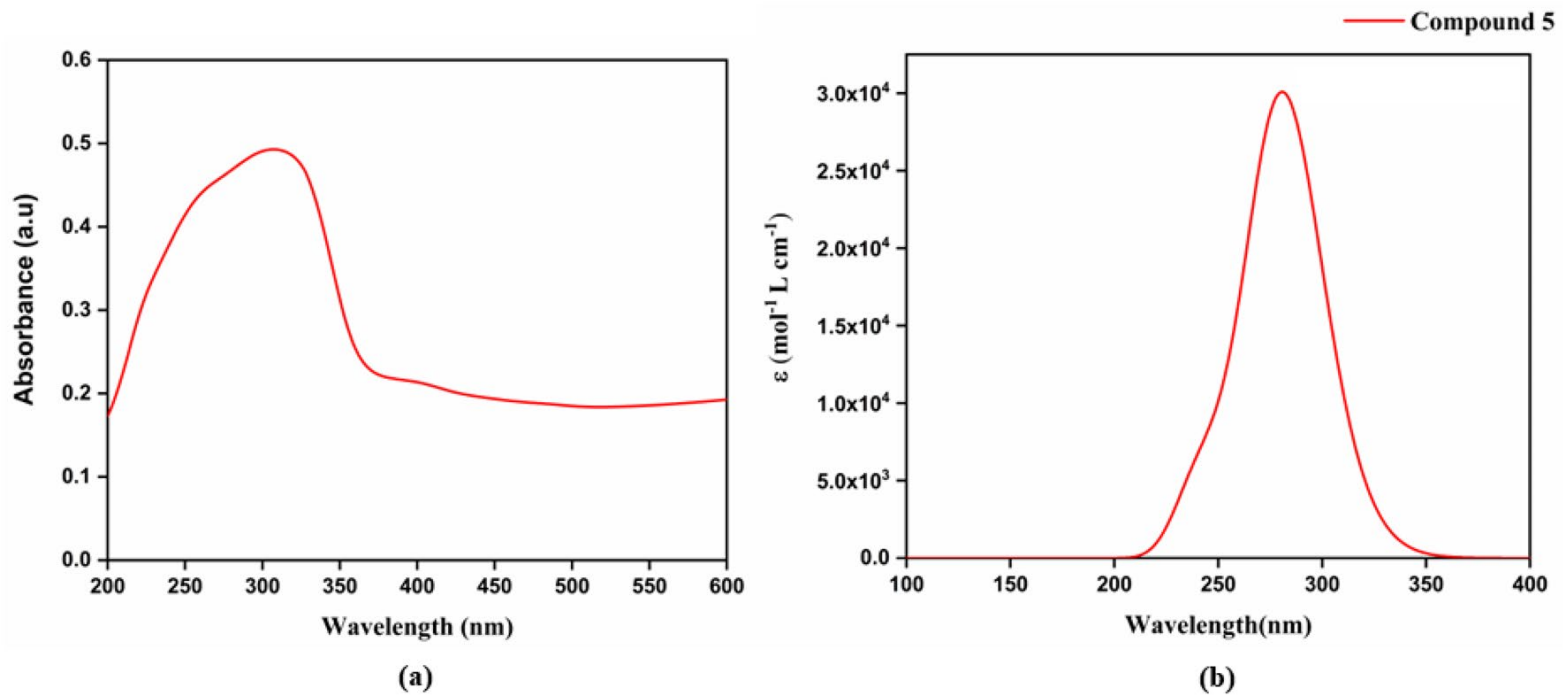
UV Visible spectrum of compound 5 from experimental (**a**) and theoretical (**b**).

UV–vis diffuse reflectance spectroscopy (UV-DRS) of compound **5** was recorded with BaSO_4_ as a reference. The compound **5** was analyzed in the range of 200–2500 nm and showed a good response in the 200–600 nm range. The λ_max_ of compound **5** is identified to be 304 nm from the UV-DRS measurements. On the other hand, using the TD-DFT calculation, we theoretically predicted the excited state behaviour of compound **5**. The quantum chemical calculations revealed absorption maxima at 280 nm, with a molar absorptivity of 3.0 × 10^4^. The TD-DFT calculations are done in a gaseous phase, which can differ from the UV-DRS absorption readings taken in a solid phase due to the differences in their respective electronic structures. In the gaseous phase, molecules are subject to different vibrational and thermal energies compared to the condensed phase. This is the reason for the significant difference in the absorption spectrum between experimental and theoretical studies. This transition is predicted from S_0_ → S_3,_ and it has an oscillator strength of 0.6151. The orbital contributions were 80% from HOMO to LUMO and the remaining 14% from HOMO-4 to LUMO. This energy gap is represented in Fig. 8 with experimental and theoretical. The TD-DFT calculation data is captured in Table 6. The HOMO-4 orbitals are mainly located around the ester bond of Compound 5, which bridges the cinnamic and nicotinic moieties. This implies that there is an intramolecular charge transfer (ICT) mechanism operating from the cinnamic moiety (donor) to the nicotinic moiety (acceptor). The efficient Donor–π–Acceptor architecture induces radiative emission capability in Compound 5. The solid-state emission property of Compound **5** is relevant in terms of organic light-emitting diode (OLED) applications. The OLED research is crucial in terms of display technology for better energy efficiency, vibrant colors, and thinner form factors.

**Table 6:** TD-DFT calculated the value for compound 5.

Name	Mol. abs (L M^−1^ cm^−1^)	Abs. max (nm)	Oscillator strength (ƒ)	Transition	Orbital contribution
Compound 5	3.0 × 10^4^	280	0.6151	S_0_ → S_3_	80% of H → L, 14% of H-4 → L,

### In silico studies for lead likeness

**Figure 9:**
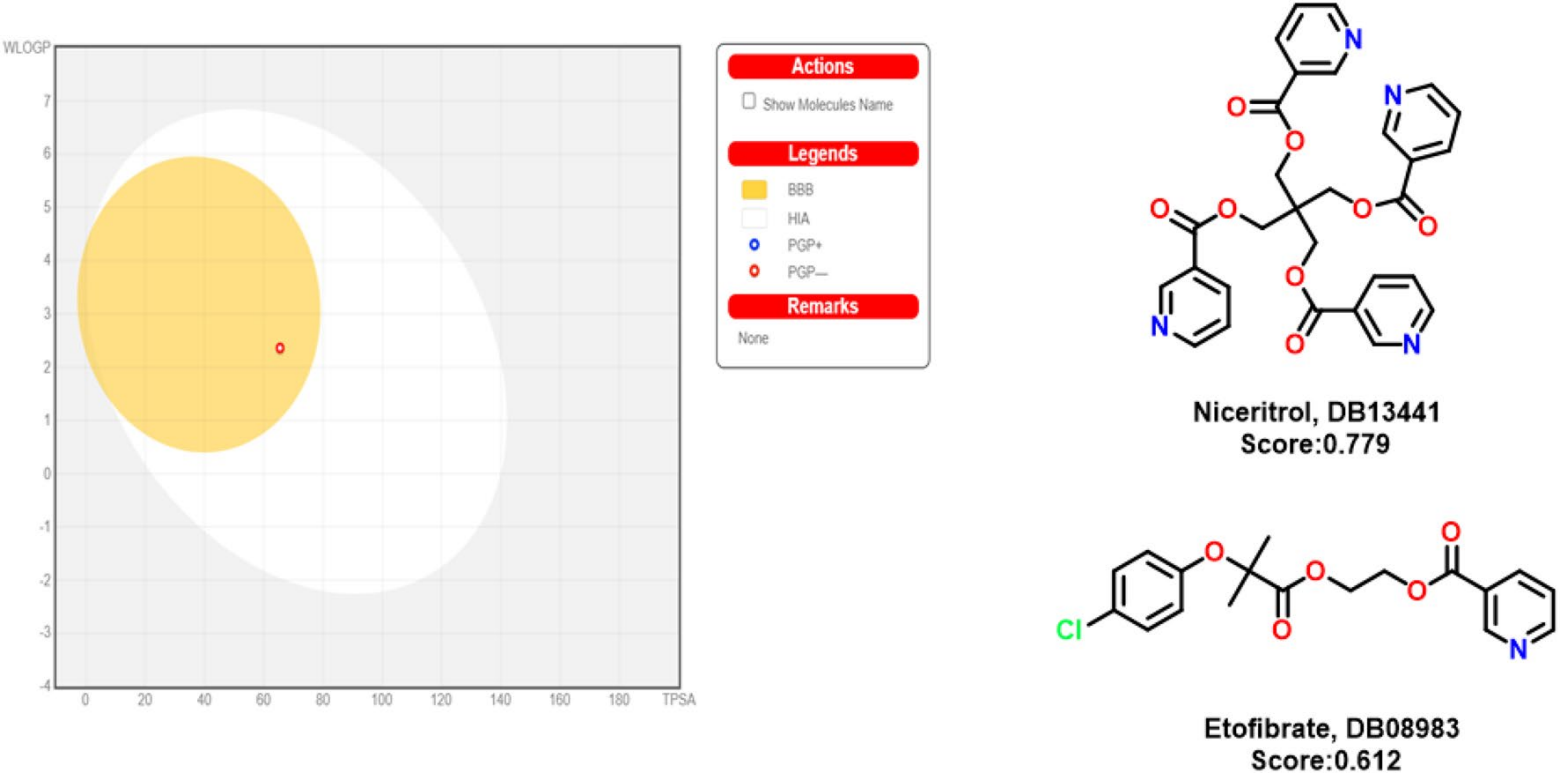
Boiled egg diagram and top two commercial drug chemical structure.

The molecule was investigated for the lead-likeness of ADMET properties; this molecule has seven rotatable bonds and five hydrogen bond acceptors. The total polar surface area is 65.49 Å^2^. The lipophilicity is 3.09 in the octanol/water system. It has moderate water solubility. This has high gastrointestinal absorption and BBB permeability. It can act as a drug for neural and other biotargets. This compound is not degraded by the liver enzymes, so the bioavailability score is noticeable. This molecule obeys the all-five drug-likeness rules, and the values are given in Table 7. The medicinal chemistry analysis data does not show any PAINS, but according to the Brenk rule, two alerts are observed. On the other hand, the synthetic availability score is 2.67. Thus, it can be easily synthesized. In the boiled egg diagram, we can see the molecule lipid permeability and BBB penetration lying in the center of region^26^. This molecule toxicity was also predicted using the pro-tox II, lying under Class 5, and the LD_50_ value of compound **5** is 2188 mg/kg^27^. This molecule shows less toxic properties, so we screened the structure using *SwissSimilarity* and analyzed the commercial drugs that have similar structures, like Compound **5** in DrugBank. The data set showed 401 molecules with score ranges between 0.779 and 0.087. The top two compounds are nicertrol and etofibrate. Figure 9 shows the boiled egg diagram and the top two commercial drug chemical structures. Both drugs have a common code: C10 (lipid modifying agents) and C (cardiovascular system). Compound **5** was screened in the ZINC database for similar bioactive compounds, with 400 compounds having a scoring range of 0.968 to 0.770. These results indicate that the molecule has the potential for biological activities^28^.

**Table 7:** Lead likeness value for the compound **5**.

Name	Rotatable bonds	H-bond acceptors	TPSA	BBB permeability	CYP1A2 inhibitor	Bioavailability score	Synthetic accessibility
Compound **5**	7	5	65.49	Yes	Yes	0.55	2.67

**Figure 10:**
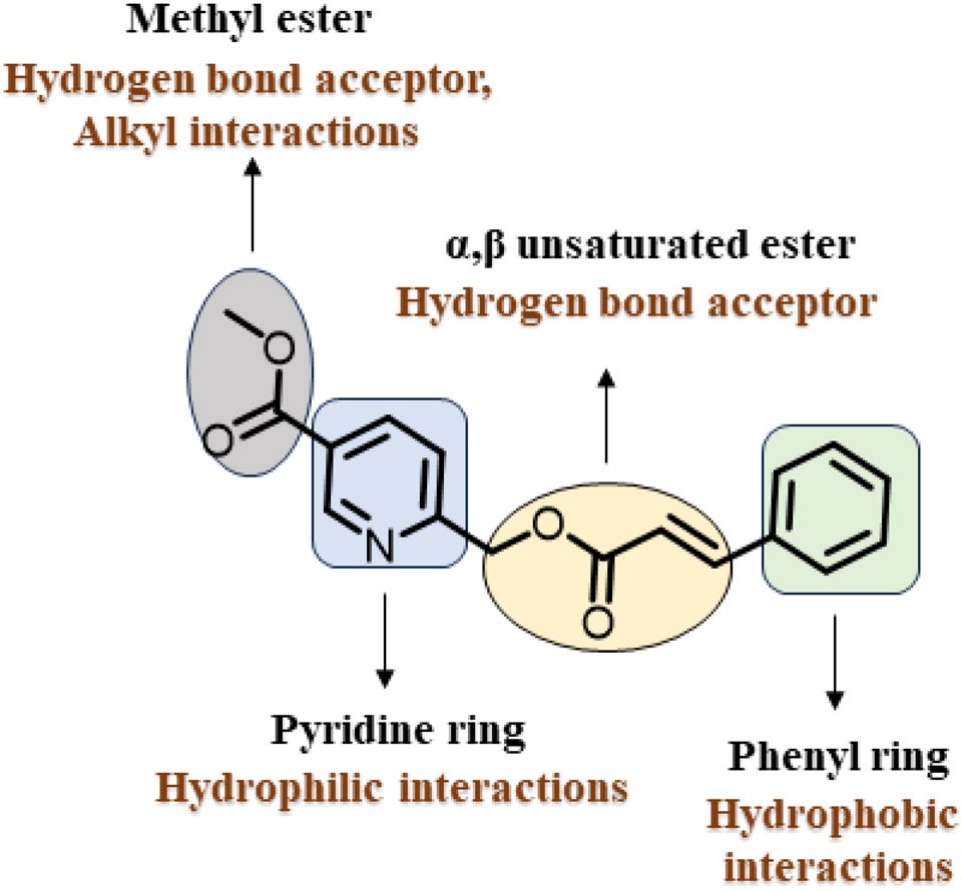
Potential interactions based on structure–activity relationship.

The protein interaction capability of the molecule is evaluated by structure-relationship (Fig. 10). The cinnamic phenyl ring provides the hydrophobic interactions, and the pyridine part provides the hydrophilic interactions. These are contributed by ring systems. The methyl ester carbonyl part acts as a hydrogen bond acceptor; methyl contributes to alkyl interactions. The linkage α,β unsaturated ester acts as a hydrogen bond acceptor.

### Molecular docking studies

**Figure 11:**
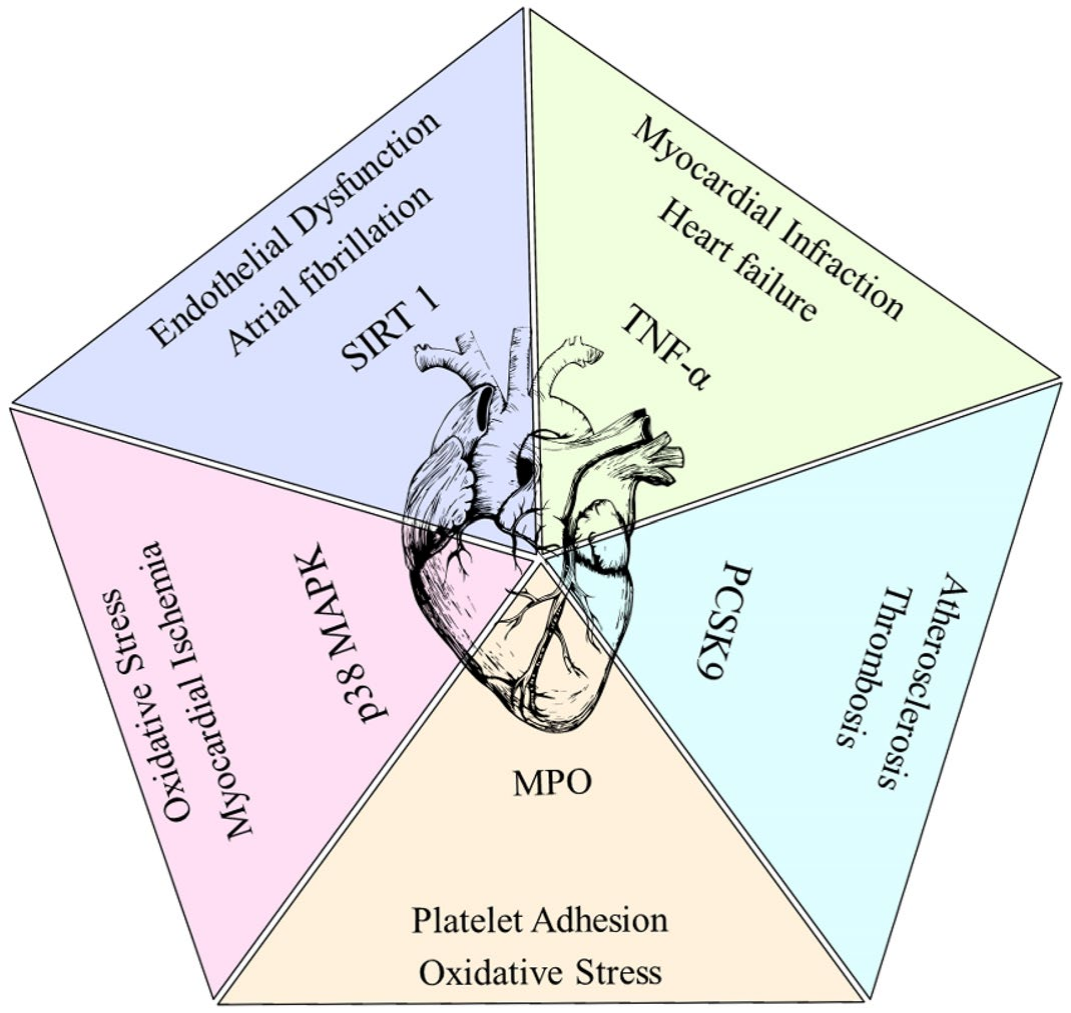
Biomarkers interlinked with cardiovascular diseases.

Based on in silico results, compound **5** has bioactive properties similar to those of the drugs that have lipid-lowering and cardiovascular protection. We have chosen the two biomarkers for the lipid pathway, which are p38 mitogen-activated kinase (MAPK) and proprotein convertase subtilisin/kexin type 9 (PCSK9). Then another two are important for cardiovascular disease, which are tumor necrosis factor alpha (TNF-α) and sirtuin 3 (SIRT3). Also, myeloperoxidase plays a major role in generating oxidative stress, which leads to cardiovascular disease (Fig. 11). The above-mentioned proteins and ligand interactions were studied by molecular docking analysis, and an optimized compound **5** structure was used as a ligand. The protein was prepared by the *Chimera X* software^29^, the previous ligands from PDB deposits were removed from the protein, and then we followed the standard docking protocol. In brief, ligand molecule charge is protected and continued with the Gasteiger charges; for protein, Kollman charges are added to the protein. The grid box was prepared, and here the blind docking protocol was followed, so the whole protein was covered by the grid box. In the parameter, the genetic algorithm is fixed, and then 100 runs are given. Then the data is processed for clustering and amino acid interactions. Then the interactions are visualized by the Discovery Studio visualizer. These data are discussed in the following sub-topics.

#### Docking of compound 5 with p38 mitogen-activated protein kinase (PDB ID:4FA2)

**Figure 12:**
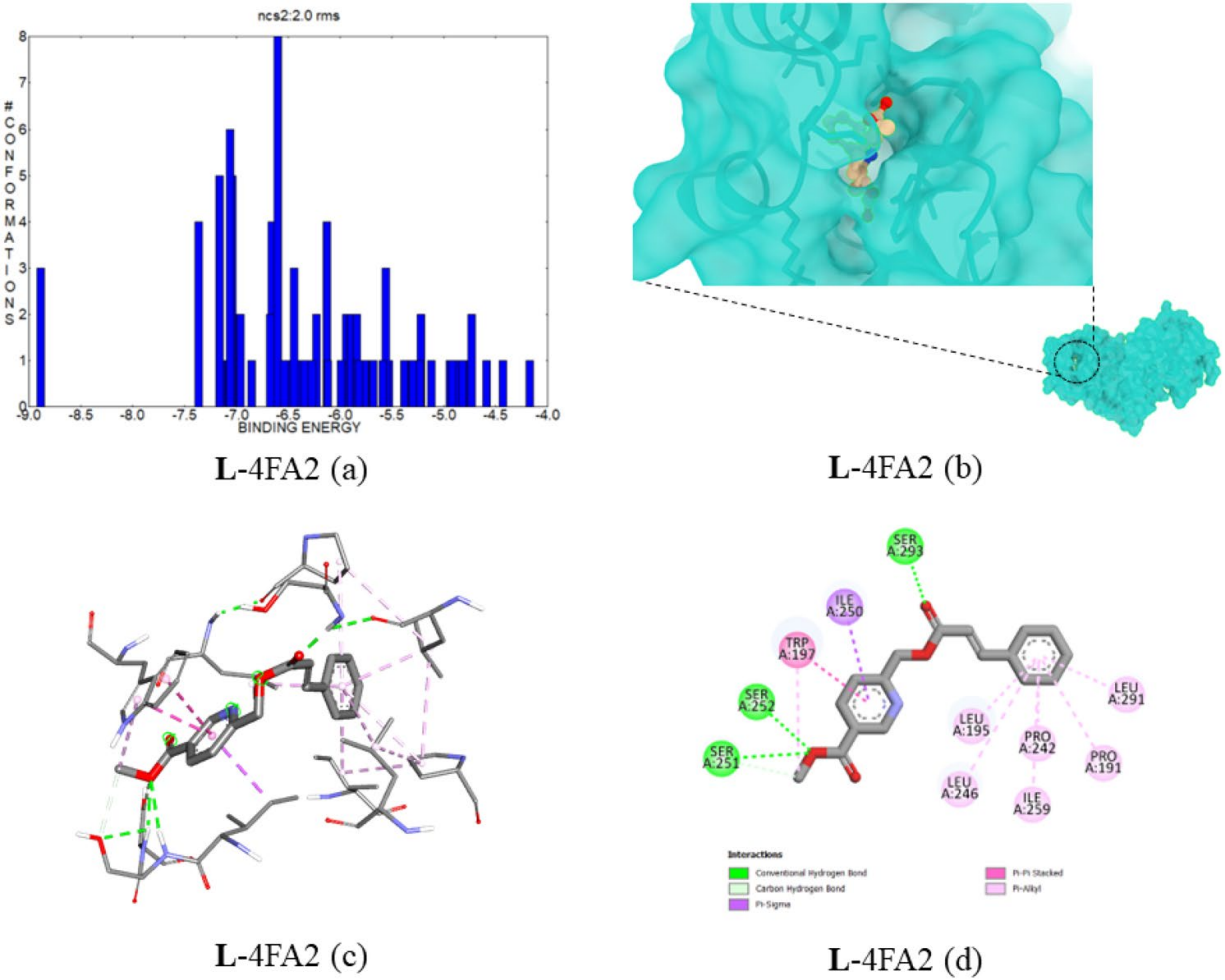
(**a**) The binding energy and number of conformations bar diagram, (**b**) ligand binding position in the pocket of protein, (**c**) 3D ligand–protein interaction, (**d**) 2D protein–ligand binding interaction with amino acids provided.

This p38 mitogen-activated protein kinase and its kinase family members are related to the inflammation and oxidative stress pathways in mammalian species^30^. This biomarker causes cardiomyocyte apoptosis, which leads to myocardial infarction. The MAPK inhibition negative consequences are none; it ensures the prominent target for our target disease^31^. The protein-structure binding interactions were examined with compound **5**. The best ligand–protein complex showed a minimum binding energy of − 8.89 kcal mol^−1^, with an estimated inhibition constant of 306.84 nM. In Fig. 12a, the cluster energies are depicted in the range of − 8.89 to − 4.1 kcal mol^−1^. Compound **5** has three conventional hydrogen bonds with SER293, SER251, and SER252. The bond distances are 1.88, 2.93, and 2.30 Å respectively. The protein–ligand binding interaction images were provided in Fig. 12b–d. The complex top five binding energies and IC_50_ values are provided in Table 8. Also, the bonding type, bonding interactions, cluster, and bond distance are provided in Table 9.

**Table 8:** Top five binding energy and IC_50_ value of compound **5** with different protein.

Complex code	Rank	Affinity (kcal/mol)	Estimated inhibition constant
Compound **5** complex with 4FA2	1	− 8.89	306.84 nM
	2	− 8.79	361.14 nM
	3	− 8.46	634.37 nM
	4	− 7.36	4.03 µM
	5	− 7.16	5.68 µM
Compound **5** complex with 2P4E	1	− 7.44	3.51 µM
	2	− 7.33	4.26 µM
	3	− 7.05	6.84 µM
	4	− 7.03	6.98 µM
	5	− 6.90	8.74 µM
Compound **5** complex with 4I5I	1	− 8.01	1.34 µM
	2	− 7.87	1.70 µM
	3	− 7.72	2.19 µM
	4	− 7.61	2.63 µM
	5	− 7.30	4.45 µM
Compound **5** complex with 2AZ5	1	− 6.22	27.60 µM
	2	− 6.17	29.80 µM
	3	− 6.15	31.17 µM
	4	− 6.09	34.41 µM
	5	− 6.06	36.26 µM
Compound **5** complex with 5FIW	1	− 6.17	29.99 µM
	2	− 5.78	57.96 µM
	3	− 5.40	110.12 µM
	4	− 5.25	141.15 µM
	5	− 5.24	143.19 µM

**Table 9:** Bonding type, bonding interactions, and bond distance for compound 5 with different proteins.

Complex code	Interaction	Distance	Bonding type
Compound **5** (L) complex with 4FA2	UNK:O3-A:SER251	2.93	Conventional hydrogen bond
	UNK:O3-A:SER252	2.30	Conventional hydrogen bond
	UNK:R2-A:TRP197	4.23	Pi–Pi stacked
	UNK:R2-A:ILE250	3.78	Pi-sigma
	UNK:O2-A:SER293	1.88	Conventional hydrogen bond
	UNK:R1-A:LEU291	4.33	Pi-alkyl
	UNK:R1-A:PRO191	5.30	Pi-alkyl
	UNK:R1-A:PRO242	4.73	Pi-alkyl
	UNK:R1-A:ILE259	5.20	Pi-alkyl
	UNK:R1-A:LEU246	5.36	Pi-alkyl
	UNK:R1-A:LEU195	4.73	Pi-alkyl
Compound **5** (L) complex with 2P4E	UNK:R1-A:VAL650	3.76	Pi-sigma
	UNK:O2-A:ARG525	1.81	Conventional hydrogen bond
	UNK:O1-A:THR459	1.89	Conventional hydrogen bond
	UNK:R2-A:THR459	2.83	Pi-lone pair
	UNK:R2-A:PRO438	5.11	Pi-ALKYL
	UNK:R2-A:ARG458	5.43	Pi-alkyl
	UNK:O3-A:VAL460	3.62	Carbon hydrogen bond
	UNK:C17-A:TRP461	3.41	Carbon hydrogen bond
	UNK:C17-A:ARG357	3.22	Carbon hydrogen bond
Compound **5** (L) complex with 4I5I	UNK:R1-A:PHE297	5.04	Pi–Pi T-shaped
	UNK:R1-A:PHE273	5.62	Pi–Pi T-shaped
	UNK:R1-A:ILE411	5.10	Pi-alkyl
	UNK:R1-A:ILE347	4.55	Pi-alkyl
	UNK:O2-A:ILE347	2.11	Conventional hydrogen bond
	UNK:R2-A:ILE270	3.75	Pi-sigma
	UNK:O4-A:GLN320	2.24	Conventional hydrogen bond
	UNK:C17-A:PHE321	4.75	Pi-alkyl
	UNK:C17-A:PRO318	5.17	Pi-alkyl
	UNK:R2-A:ILE316	4.61	Pi-alkyl
	UNK:R2-A:ILE316	2.93	Carbon hydrogen bond
Compound **5** (L) complex with 2AZ5	UNK:R1-D:LEU93	5.22	Pi-alkyl
	UNK:R1-B:LEU93	5.34	Pi-alkyl
	UNK:O1-D:GLN125	2.71	Unfavorable acceptor–acceptor
	UNK:O2-D:GLN125	1.97	Conventional hydrogen bond
	UNK:C10-B:LEU93	3.45	Carbon hydrogen bond
	UNK:R2-D:ARG82	4.18	Pi-cation
	UNK:O4-B:ASN92	2.17	Conventional hydrogen bond
	UNK:C17-B:VAL91	4.71	Alkyl
Compound **5** (L) complex with 5FIW	UNK:R1-B:MET87	5.89	Pi-sulfur
	UNK:R1-B:GLY90	3.77	Amide-Pi stacked
	UNK:O2-B:HIS95	3.19	Carbon hydrogen bond
	UNK:O1-D:HIS336	1.98	Conventional hydrogen bond
	UNK:R2N-B:GLN91	2.06	Conventional hydrogen bond
	UNK:R2-D:GLU242	3.63	Pi-Anion
	UNK:R2-D:ARG239	5.01	Pi-Alkyl
	UNK:C17-D:PHE366	5.45	Pi-Alkyl
	UNK:C17-D:PHE407	5.38	Pi-Alkyl

#### Docking of compound 5 with proprotein convertase subtilisin/kexin type 9 (PCSK9) (PDB ID:2P4E)

**Figure 13:**
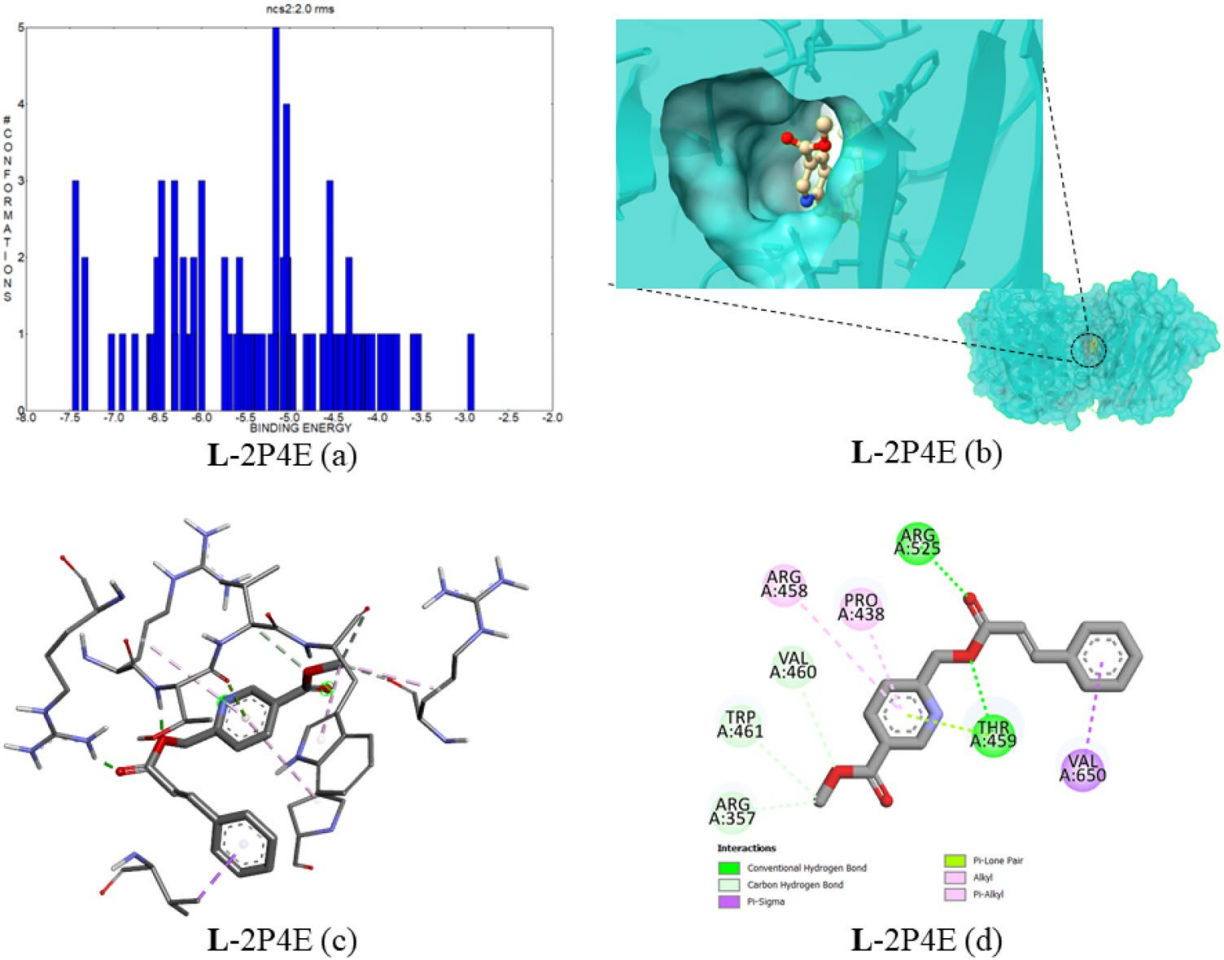
(**a**) The binding energy and number of conformations bar diagram, (**b**) ligand binding position in the pocket of protein, (**c**) 3D ligand–protein interaction, (**d**) 2D protein–ligand binding interaction with amino acids provided.

PCSK9 is directly related to dyslipidemia and low-density lipoprotein (LDL) receptors; it causes the degradation of LDL receptors, thereby restricting LDL removal from the blood circulation. It ends with threatening cardiovascular events^32,33^. This PCSK9 inhibition needs to be investigated through ligand–protein interactions. The binding complex of Compound **5** with 2P4E, the best ligand–protein complex, showed a score of − 7.44 kcal mol^−1^, with an estimated inhibition constant of 3.51 µM. In Fig. 13a, the cluster energies are depicted in the range of − 7.44 to − 3.44 kcal mol^−1^. In that ligand–protein complex, the ligand has two conventional hydrogen bonds with ARG525 and THR459; the bond distance is 1.81 and 1.89 Å, respectively. The protein–ligand binding interaction images are provided in Fig. 13b–d. The complex top five binding energies and IC_50_ values are provided in Table 8. Also, the bonding type, bonding interactions, cluster, and bond distance are provided in Table 9.

#### Docking of compound 5 with Sirtuin 1 (SIRT1) (PDB ID:4I5I)

**Figure 14:**
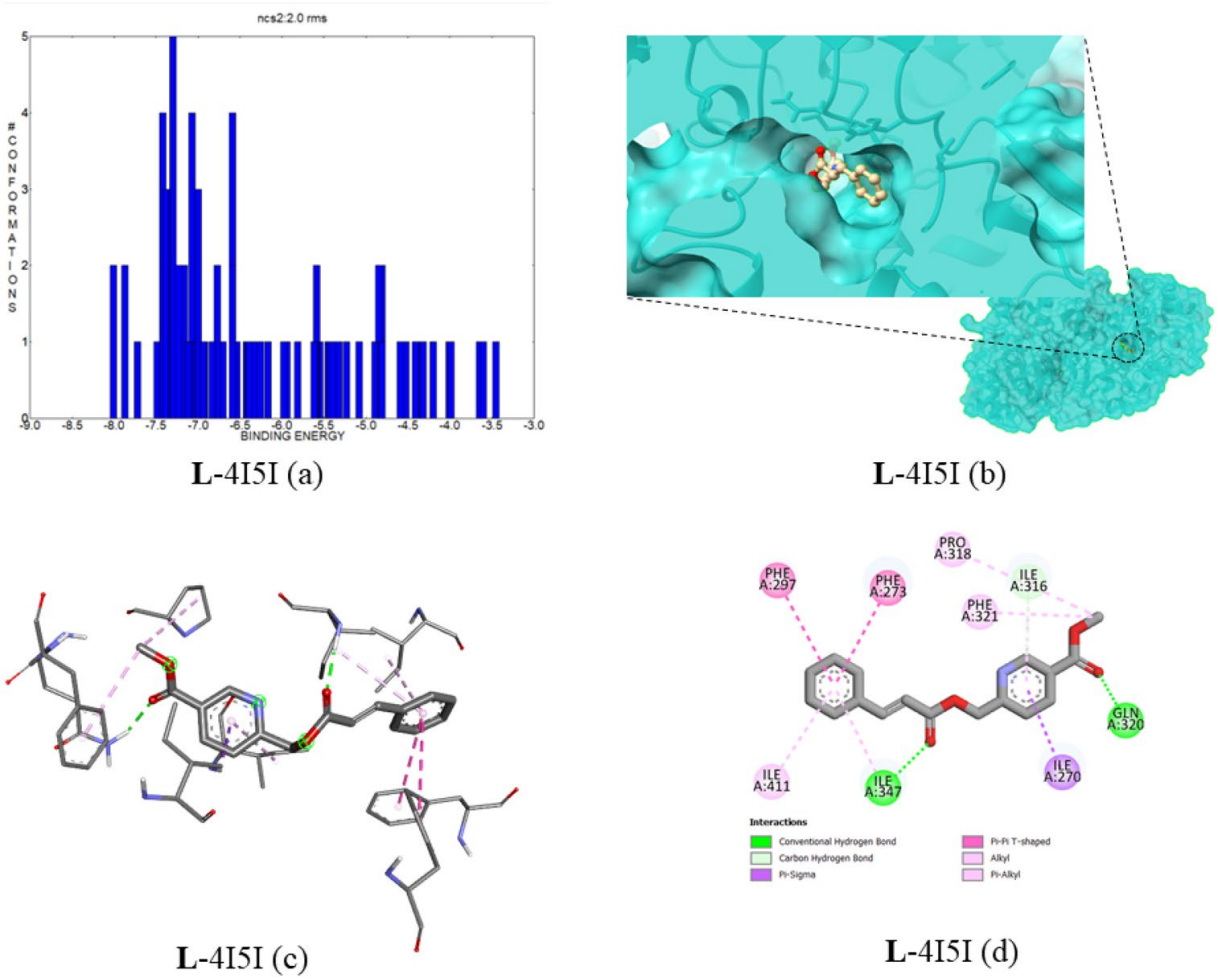
(**a**) The binding energy and number of conformations bar diagram, (**b**) ligand binding position in the pocket of protein, (**c**) 3D ligand–protein interaction, (**d**) 2D protein–ligand binding interaction with amino acids provided.

The Sirtuin protein family involves various biological processes; through its catalytic action on the reversible deacylation of lysines inside the Notch 1 intracellular domain, SIRT1 adversely controls Notch signaling in endothelial cells. Also, this gene expression polymorphism increases the possibility of CVD, and this marker has benefits for aging-related cardiac therapy^34–36^. Compound **5** is docked with the SIRT1 protein; in this case, the best binding interaction of ligand–protein was observed at − 8.01 kcal mol^−1^, with an estimated inhibition constant value of 1.34 µM. In Fig. 14a, the cluster interaction energies are depicted in the range of − 8.01 to − 3.04 kcal mol^−1^. The two conventional hydrogen bonds are formed by ligand with the amino acid residues of ILE347 and GLN320 and have a bond distance of 2.11 and 2.44 Å, respectively. The protein–ligand binding interaction images are provided in Fig. 14b–d. The complex top five binding energies and IC_50_ values are provided in Table 8. Also, the bonding type, bonding interactions, cluster, and bond distance are provided in Table 9.

#### Docking of compound 5 with Tumour necrosis factor alpha (TNF-α) (PDB ID:2AZ5)

**Figure 15:**
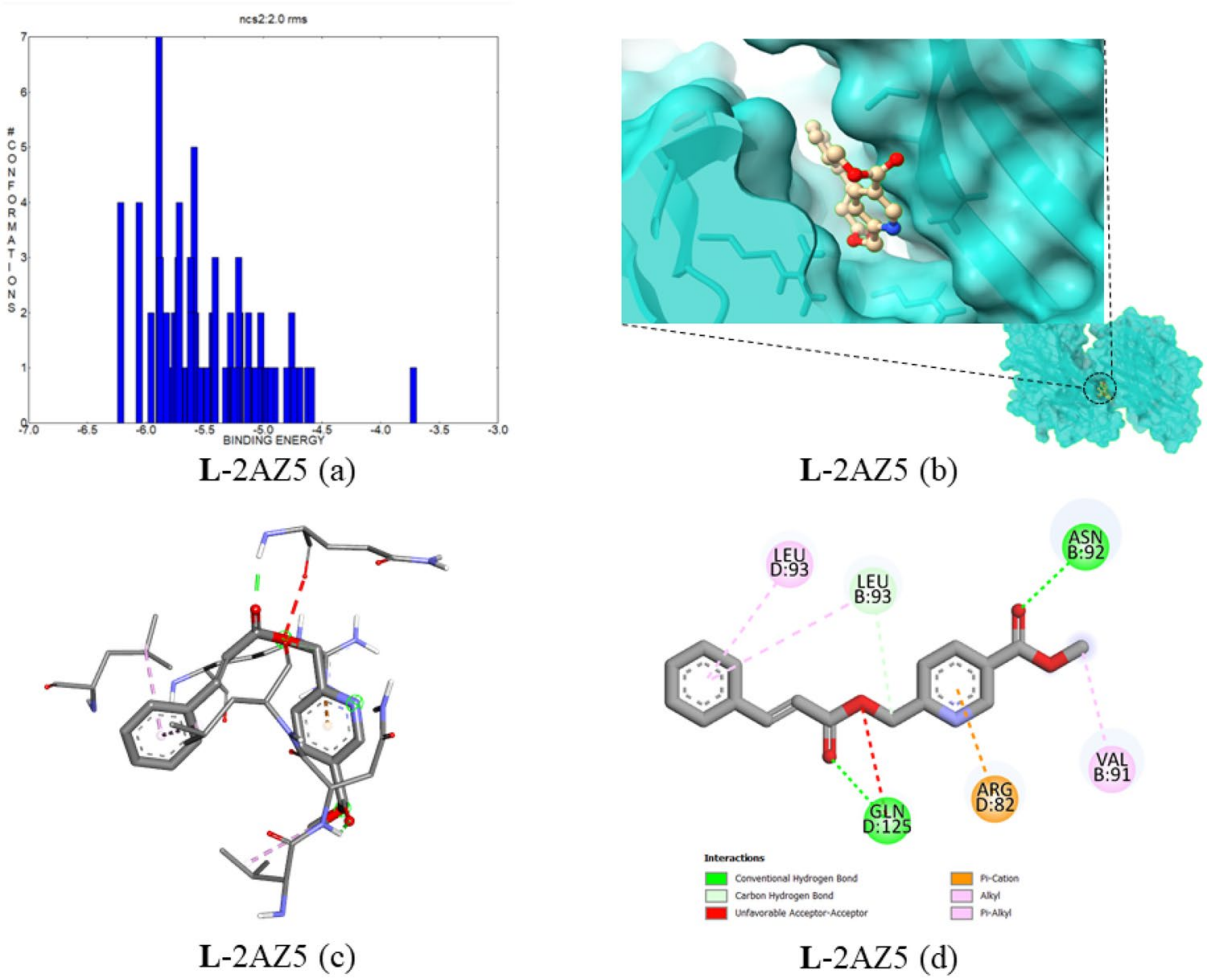
(**a**) The binding energy and number of conformations bar diagram, (**b**) ligand binding position in the pocket of protein, (**c**) 3D ligand–protein interaction, (**d**) 2D protein–ligand binding interaction with amino acids provided.

TNF-α protein controls vascular permeability; abnormal levels of expression cause an increased level of permeability due to this subendothelial accumulation of blood lipids and inflammatory cells. This forms the atherosclerotic plaques in arteries^37^. Some other non-communicable diseases, because of these characteristics, are considered a promising target^38^. Compound **5** is docked with the TNF-α protein, and the best binding interaction value is − 6.22 kcal mol^−1^; the estimated inhibition constant is 27.60 µM. In Fig. 15a, the cluster interaction energies are depicted in the range from − 6.22 to − 4.6 kcal mol^−1^, which has two covalent hydrogen bonds with the amino acid residues ASN92 and GLN125 with bond distances of 2.17 and 1.97 Å, respectively. The protein–ligand binding interaction images were provided in Fig. 15b–d. The complex top five binding energies and IC_50_ values are provided in Table 8. Also, the bonding type, bonding interactions, cluster, and bond distance are provided in Table 9.

#### Docking of compound 5 with myeloperoxidase (MPO) (PDB ID:5FIW)

**Figure 16:**
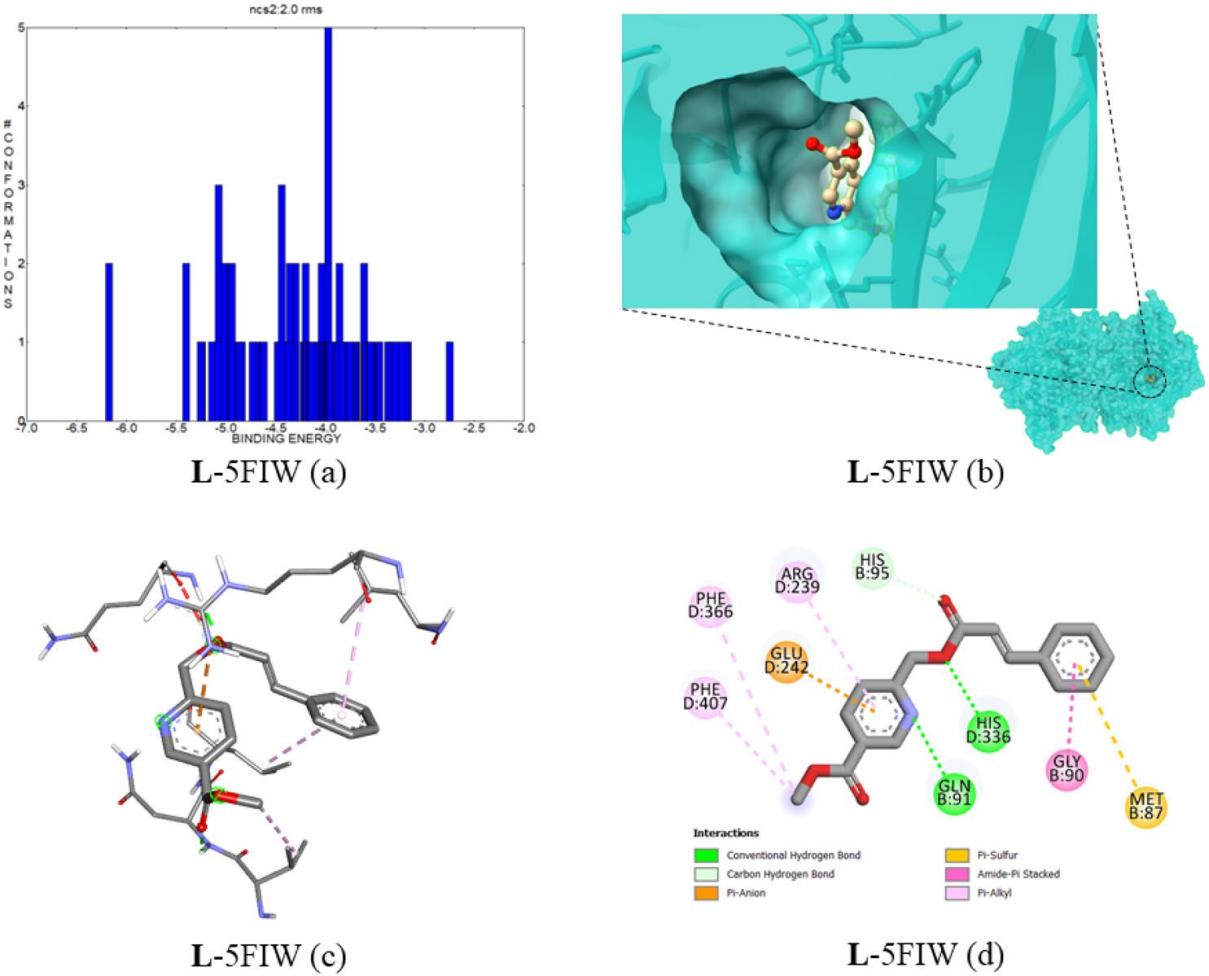
(**a**) The binding energy and number of conformations bar diagram, (**b**) ligand binding position in the pocket of protein, (**c**) 3D ligand–protein interaction, (**d**) 2D protein–ligand binding interaction with amino acids provided.

MPO undergoes the halogenation cycle to produce harmful hypohalous acid; further free radicals are produced by the one-electron process and activated oxygen. It oxidizes the HDL protein; this Ox-HDL leads to critical cardiovascular events^39,40^. It is also linked with various diseases, including Parkinson’s and rheumatoid arthritis^41^. The MPO protein is docked with Compound **5**, with the best binding interaction of − 6.17 kcal mol^−1^, and the estimated inhibition is 29.99 µM. In Fig. 16a, the cluster interaction energies are depicted in the range of − 6.17 to − 3.2 kcal mol^−1^. The ligand has two conventional hydrogen bonds with the protein amino acid residues GLN91 and HIS336 with a distance of 2.06 and 1.98 Å, respectively. The protein–ligand binding interaction images were provided in Fig. 16b–d. The complex top five binding energies and IC_50_ values are provided in Table 8**.** Also, the bonding type, bonding interactions, cluster, and bond distance are provided in Table 9.

### MTT cell viability assay

**Figure 17:**
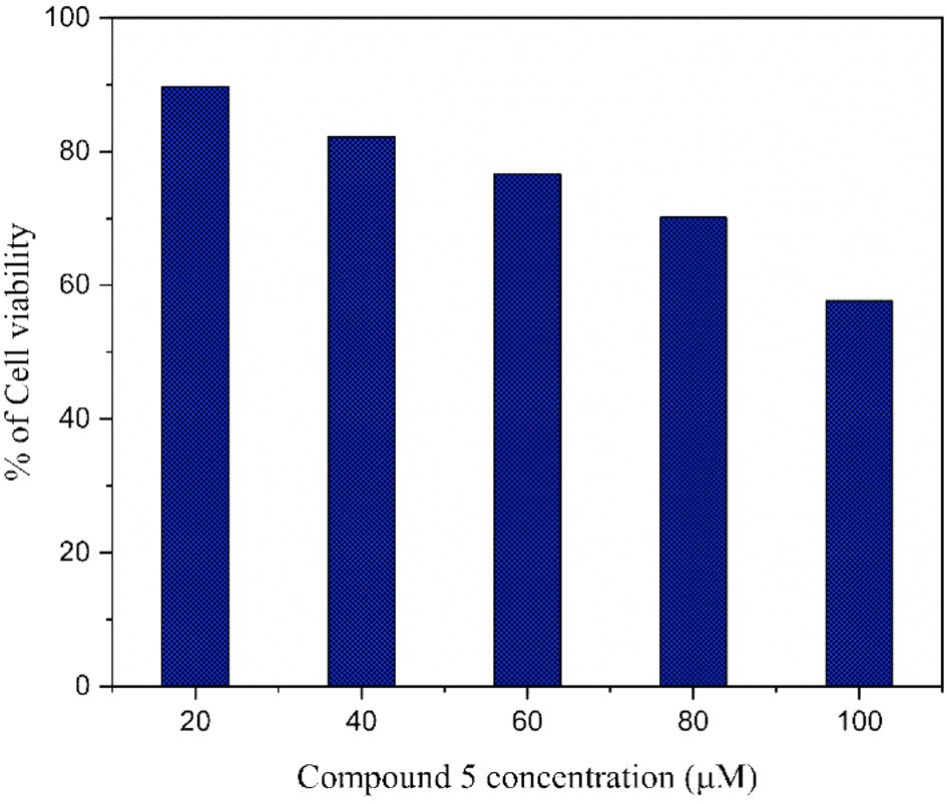
Results of MTT cell viability assay.

The toxicity of compound **5** was investigated by the MTT cell viability assay. Which is performed with RAW-264.7 macrophage cells in different concentrations of synthesized molecules. The outcome (Table S1, Fig. 17) of the study established Compound **5** is preferable for in vivo studies. The outcome of the results denoted that phytochemical-based conjugates have less toxicity.

### Stability study in different temperatures

Compound **5** was kept at different temperatures to evaluate the transient behavior of the α,β unsaturated ester bond. It can easily undergo thermal degradation, which affects the shelf life of the molecule. The substance was stored for 48 h at three different temperatures: 4 °C, 33 °C, and 37 °C, respectively. After that, the substance was dissolved in the ethyl acetate, and the samples were compared with the previous step's starting materials by using the thin-layer chromatography method with a 20% ethyl acetate and hexane solvent mixture. Compound **5** shows (Fig. S10) the spots with the same retention factor value for the different temperature samples, and no other spots are also noticed. The acid and alcohol functional groups containing starting materials have different retention factor values. From this, compound **5** will not undergo the thermal degradation of the α, β unsaturated ester bond. Which can increase the possibilities for the shelf life of the lead molecule.

## Conclusion

Cinnamoyl picolinate (compound **5**) was synthesized and characterized by various analytical spectroscopic techniques. The three-dimensional characteristics of the molecule were established by single-crystal analysis which was being used for theoretical studies, generating surface maps, and fingerprint maps. Two intermolecular hydrogen bonding interactions were observed, with values of 2.406 and 3.227 Å, respectively. Notably, these interactions seemed to increase the possibility of cinnamoyl picolinate interacting with the biotargets such as …….. For better structure optimization, in-depth theoretical studies such MEP maps, TD-DFT, and FMO analysis were undertaken. The experimentally optimized structure, bond angles, and bond distances were compared with SC-XRD data. From the MEP data, the nucleophilic and electrophilic regions have been identified and their corresponding molecular electrostatic potential values were from − 0.05094 to 0.05094 a.u. The theoretical TD-DFT UV spectrum was compared with the experimental UV spectrum. We have also examined the lead-likeness properties of compound 5 by performing in silico studies and the results seemed to satisfy all drug-likeness rules. The parent compound 5 was analyzed for structural similarity with the existing drugs available in the market with specific bio-targets. It showed promising interactions and calculated inhibition constants were at nano- and micromolar concentration levels. Stability and viability results revealed that the molecule is suitable for further development. Therefore, cinnamoyl picolinate is a potent lead molecule for MAP kinase and it can be prepared from natural source with minimal cost.

### Supplementary Information

Supplementary Information.

## Data Availability

We declare that supporting data for this work is provided in the Supplementary Information. Raw and unprocessed nuclear magnetic resonance and other data are available from the corresponding author on reasonable request. Compound 5 crystallographic data is available free of charge from the Cambridge Crystallographic Data Centre under deposition number CCDC2221434. Website: https://www.ccdc.cam.ac.uk/structures.
