# Supplementary material for: Development of methyl 5-((cinnamoyloxy)methyl)picolinate, exploring its bio-target potential aiming at CVD mediated by multiple proteins through surface and physiochemical analysis
Source: Sci Rep. 2024 Jun 10;14:13328. doi: 10.1038/s41598-024-64165-7 (PMC11164877; doi:10.1038/s41598-024-64165-7)
Supplement: Supplementary file 1 — Supplementary Information. [file 41598_2024_64165_MOESM1_ESM.docx]

**Development of methyl 5-((cinnamoyloxy)methyl)picolinate, exploring its bio-target potential aiming at CVD mediated by multiple proteins through surface and physiochemical analysis**

**Lenin Nachimuthu and Rajagopal Desikan***

*Department of Chemistry, School of Advanced Sciences (SAS), Vellore Institute of Technology (VIT), Vellore, India.*

**Corresponding author email ID:* [*rajagopal.desikan@vit.ac.in*](mailto:rajagopal.desikan@vit.ac.in) *(Rajagopal D*

**Electronic Supplementary Materials**


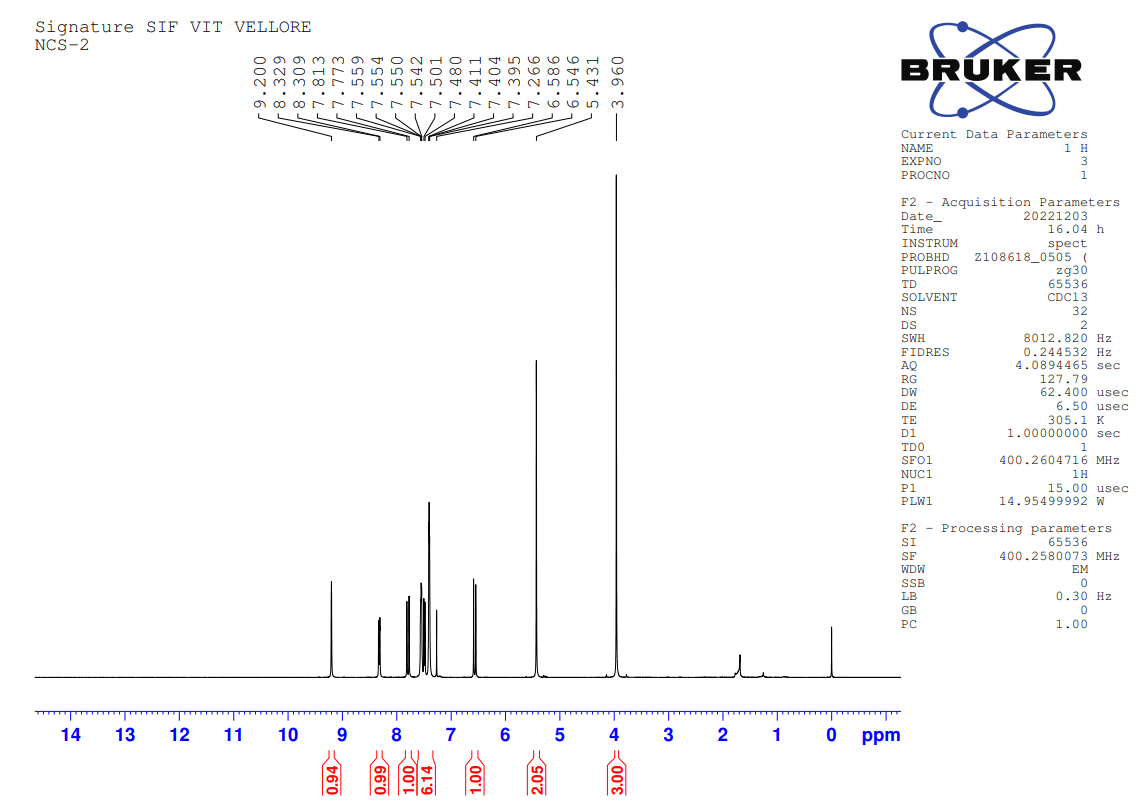


**Figure S1**: ^1^H NMR spectrum of Compound 5


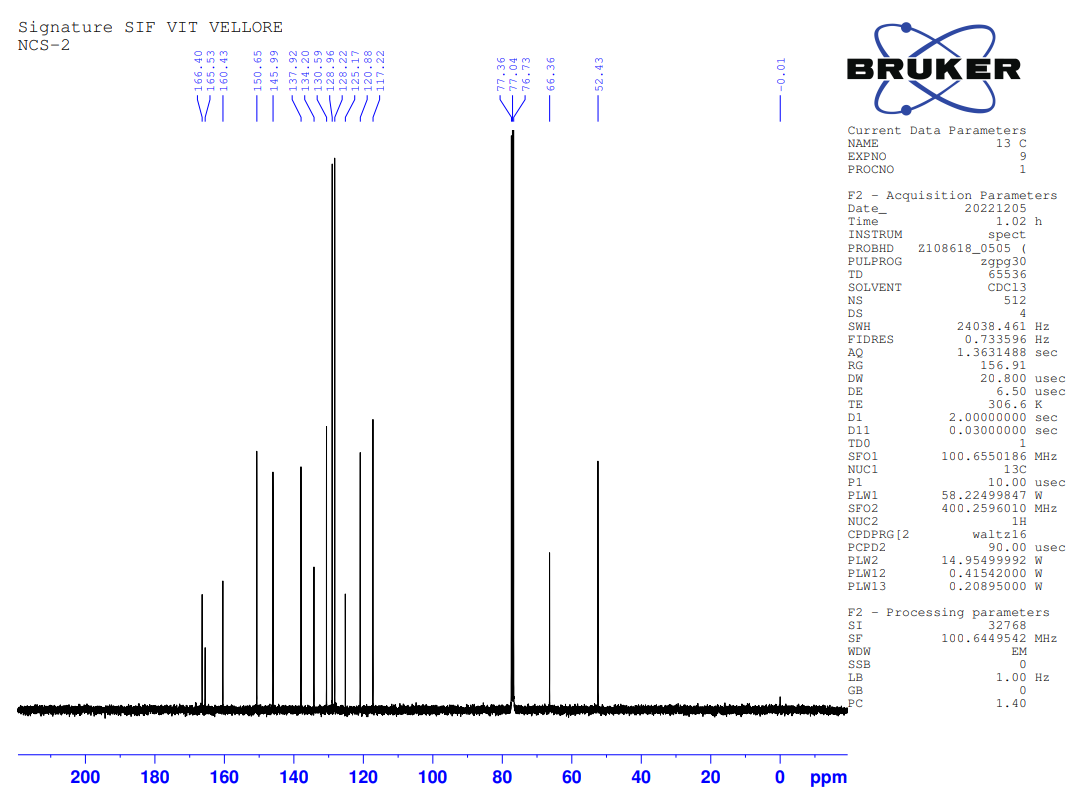


**Figure S2**: ^13^C NMR spectrum of Compound 5


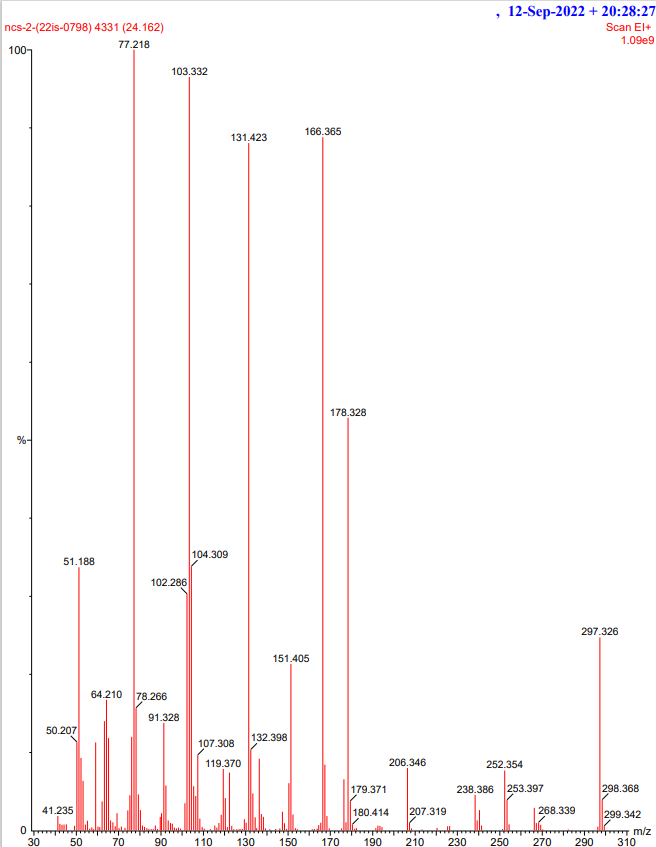


**Figure S3**: GC-MS spectrum of compound


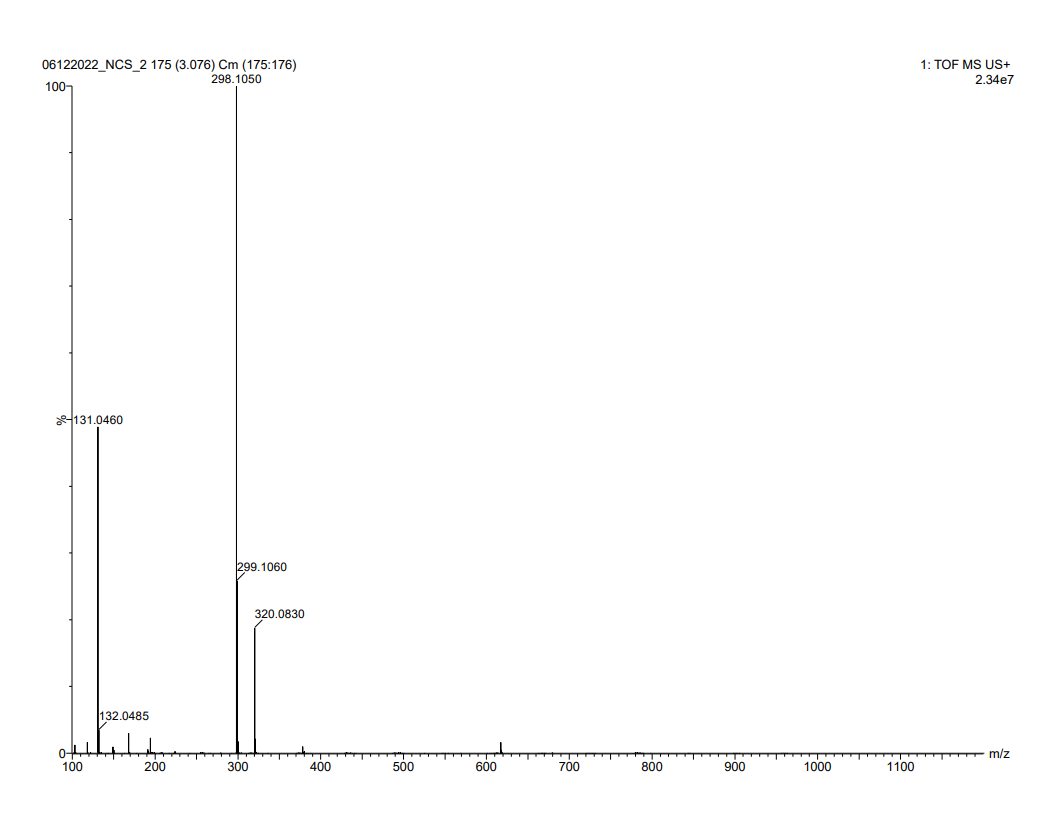


**Figure S4**: HRMS spectrum of compound


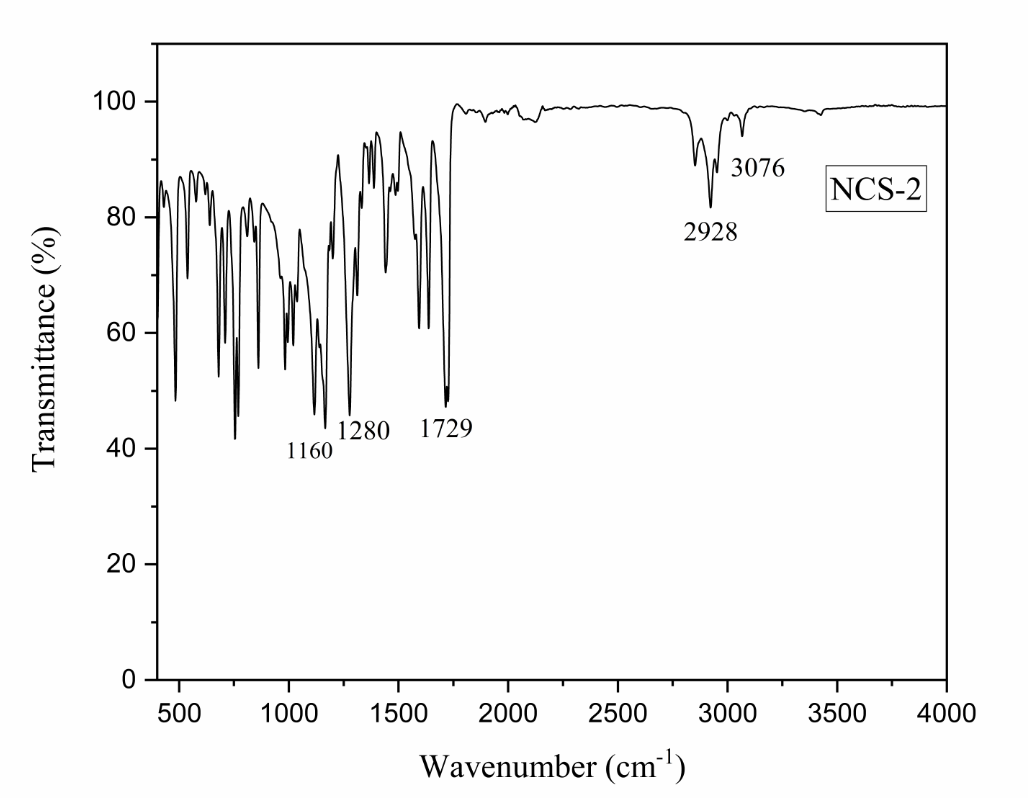


**Figure S5**: FT-IR spectrum of Compound


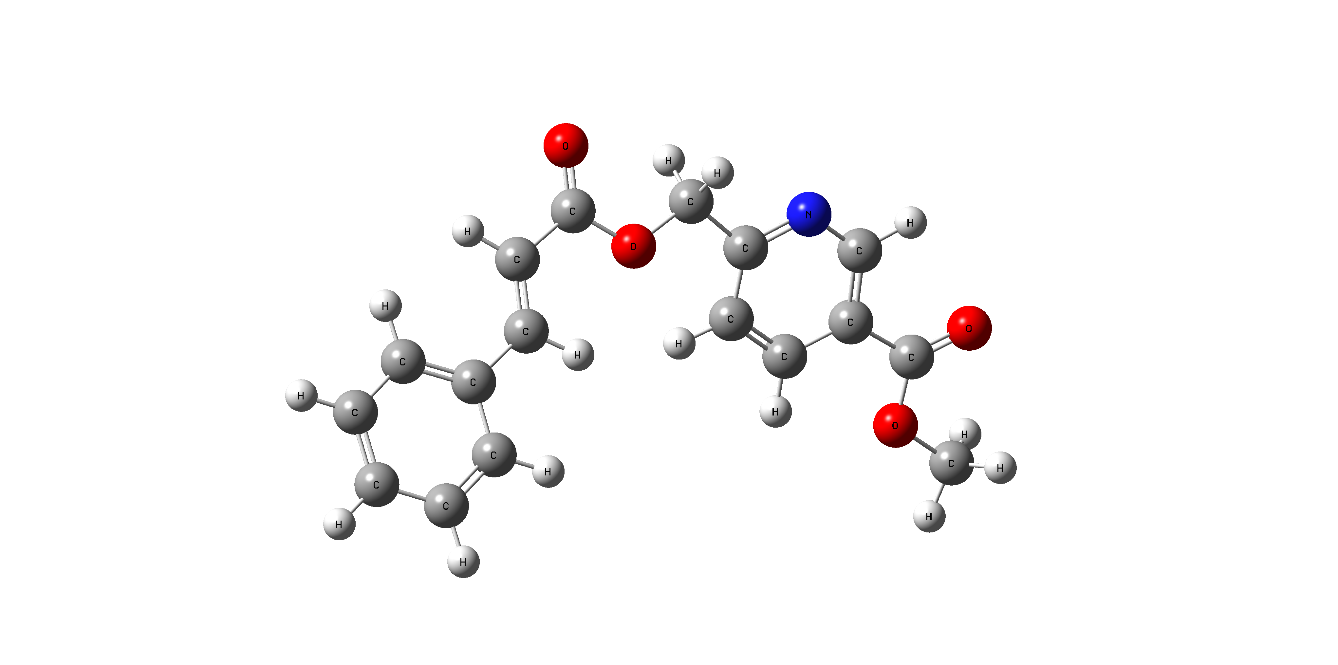


**Figure S6:** optimized structure of Compound **5**


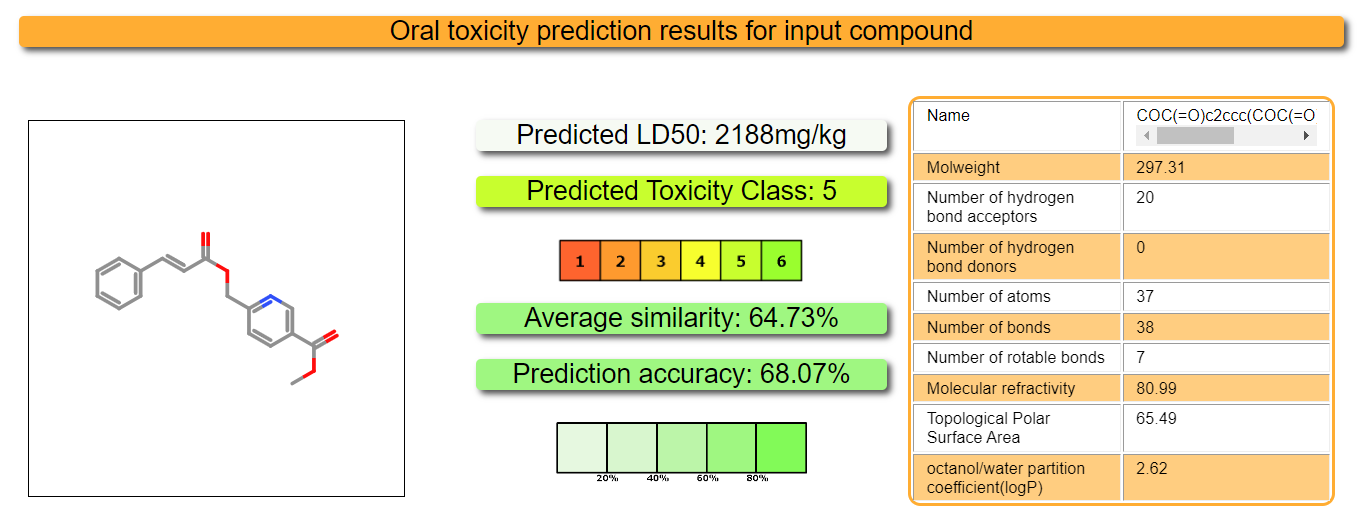


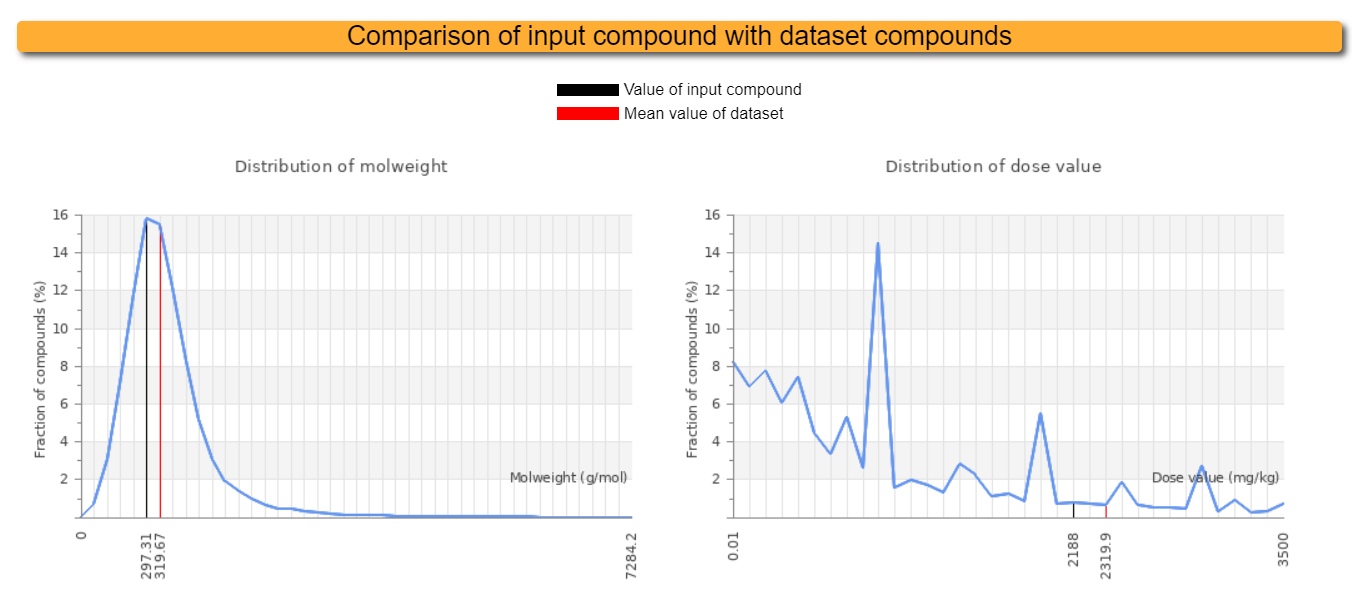


**Figure S7:** Predicted toxicity results by Protox II.

**
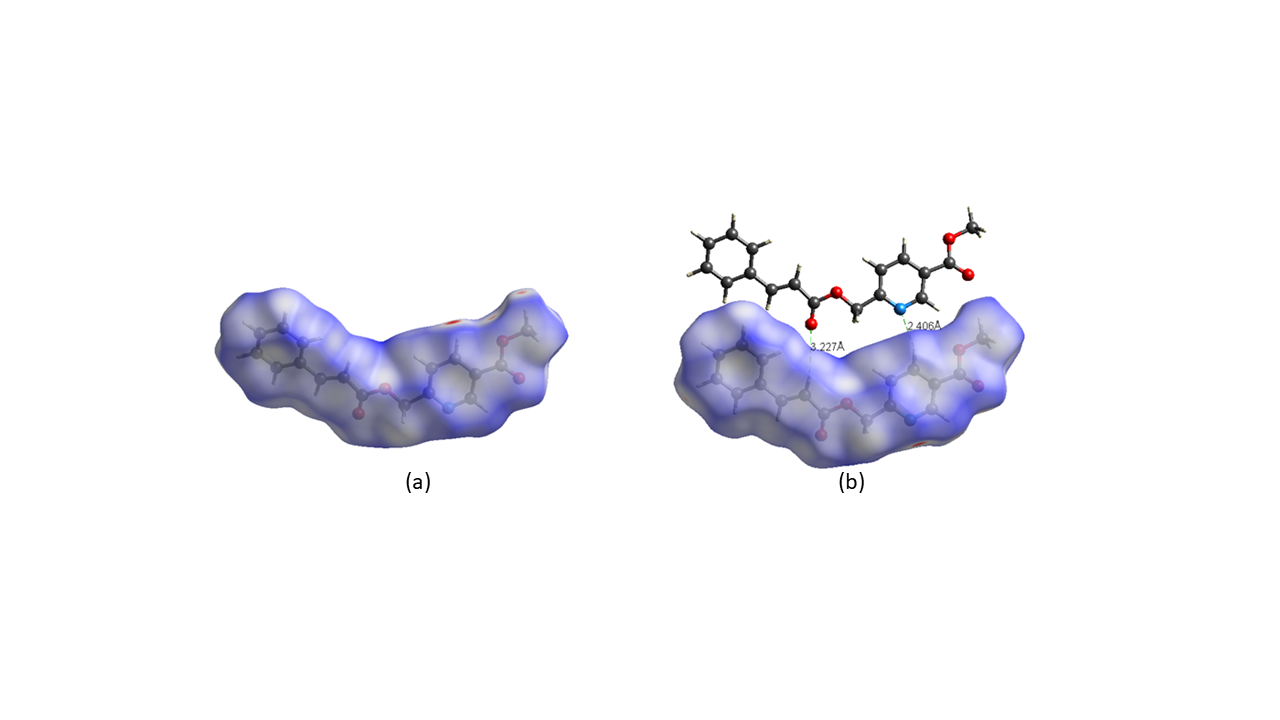
**

**Figure S8. (a)**d_norm_ and **(b)** d_norm_ Interactions

**Figure S9.** GC-MS molecular fragmentation of compound-5 and its structural visualization with mass value


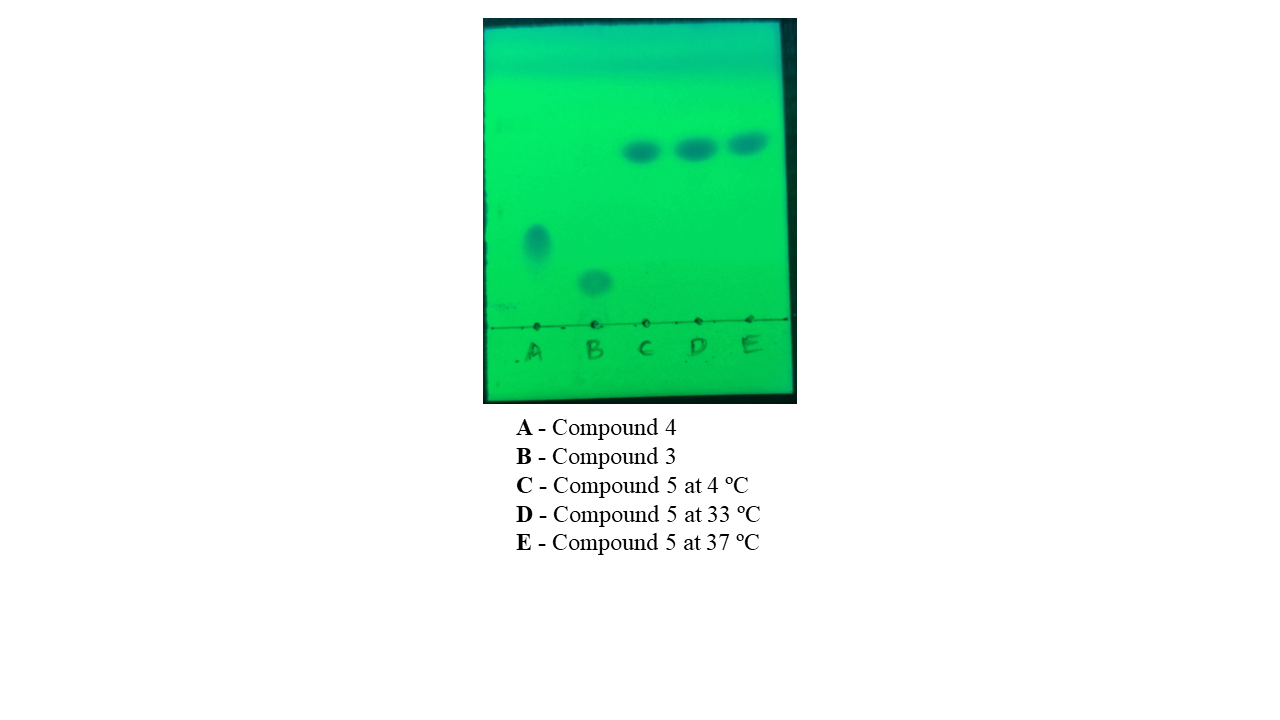


**Figure S10.** Stability analysis of the compound **5** in various temperatures by thin layer chromatography method.

**1.MTT assay protocol:**

In a 96-well plate, 1x105 cells/ml were seeded and kept for 24 hours at 37 °C in 5% CO2. Subsequently, different concentrations of compound **5** were applied to the cells. The treated cells were then incubated for a further 24 hours at 37 °C in an incubator with 5% CO2. Then each well was filled with approximately 50 μL of MTT dye, and it was incubated for two hours. After that, it was solubilized in DMSO, and a multimode varioscan (Thermo Scientific) was used to detect the absorbance at 570 nm.

**Table S1.** Results of Compound **5** with different concentrations and RAW macrophage cell line

| Compound **5** concentration  (µM) | % of Cell viability |
| --- | --- |
| Control | 100 |
| 20 | 89.74 |
| 40 | 82.16 |
| 60 | 76.58 |
| 80 | 70.12 |
| 100 | 57.63 |
